# Future bioenergy expansion could alter carbon sequestration potential and exacerbate water stress in the United States

**DOI:** 10.1126/sciadv.abm8237

**Published:** 2022-05-04

**Authors:** Yanyan Cheng, Maoyi Huang, David M. Lawrence, Katherine Calvin, Danica L. Lombardozzi, Eva Sinha, Ming Pan, Xiaogang He

**Affiliations:** 1 Department of Industrial Systems Engineering and Management, National University of Singapore, Singapore, Singapore.; 2 Atmospheric Sciences and Global Change Division, Pacific Northwest National Laboratory, Richland WA, USA.; 3 Climate and Global Dynamics Laboratory, National Center for Atmospheric Research, Boulder, CO, USA.; 4 Joint Global Change Research Institute, Pacific Northwest National Laboratory, Riverdale Park, MD, USA.; 5 CW3E, Scripps Institution of Oceanography, University of California San Diego, San Diego, CA, USA.; 6 Department of Civil and Environmental Engineering, National University of Singapore, Singapore, Singapore.

## Abstract

The maximum future projected bioenergy expansion potential, in scenarios limiting warming to 2°C or below, is equivalent to half of present-day croplands. We quantify the impacts of large-scale bioenergy expansion against re/afforestation, which remain elusive, using an integrated human-natural system modeling framework with explicit representation of perennial bioenergy crops. The end-of-century net carbon sequestration due to bioenergy deployment coupled with carbon capture and storage largely depends on fossil fuel displacement types, ranging from 11.4 to 31.2 PgC over the conterminous United States. These net carbon sequestration benefits are inclusive of a 10 PgC carbon release due to land use conversions and a 2.4 PgC loss of additional carbon sink capacity associated with bioenergy-driven deforestation. Moreover, nearly one-fourth of U.S. land areas will suffer severe water stress by 2100 due to either reduced availability or deteriorated quality. These broader impacts of bioenergy expansion should be weighed against the costs and benefits of re/afforestation-based strategies.

## Teaser

A human-Earth system model framework is developed to assess the impact of BECCS and reforestation on CO_2_ removal and water stress.

## INTRODUCTION

Bioenergy with carbon capture and storage (BECCS) is a key carbon removal strategy in future socioeconomic pathways that are designed to limit global warming to well below 2°C by the end of the 21st century under the Paris Agreement (*1*–*5*). For example, future global land areas for bioenergy deployment range from 1.4 to 7.5 million ﻿km^2^ in scenarios limiting warming to 2°C from the SR1.5 database (Special Report on Global Warming of 1.5°C) (*6*). Estimates across 15 future scenarios, combining the Shared Socioeconomic Pathways (SSPs) with different Representative Concentration Pathways (RCPs) (*4*, *7*–*10*), project that 2.8 to 17.8 million km^2^ of global land will be devoted to second-generation bioenergy crops (switchgrass and *Miscanthus*) by 2100. Over the contiguous United States (CONUS), the projected plantation area of bioenergy crops would occupy 0.3 to 1.9 million km^2^ of land by 2100, equivalent to 13 to 89% of the cropland area in 2015 (*11*).

Despite the carbon removal benefits of BECCS (*12*), emerging studies (*3*, *13*, *14*) find that bioenergy crop expansion can strongly modulate land surface dynamics and lead to negative impacts on terrestrial biogeochemical and hydrologic budgets, such as increased land-based carbon emissions (*2*, *15*), changes in the hydrologic cycle (*16*) including enhanced water stress (*13*, *17*), and deteriorated water quality associated with increased nitrogen loading in rivers (*18*), as bioenergy expansion is associated with large-scale land use changes and the high productivity of bioenergy crops across a long growing season substantially increases water consumption (*16*). However, most of these assessments have focused on relatively small domains or have ignored crucial carbon-water-nitrogen interactions. Assessments of water stress should account for both water availability and water quality because the usability of water depends on both sufficient quantity and suitable quality (*19*). Meanwhile, impact of bioenergy expansion on carbon removal and water supply should be jointly assessed given the intricate linkage between biogeochemical and hydrological processes. In addition, an alternative carbon sequestration strategy is re/afforestation. Despite a growing body of research (*2*, *20*) on these land-based carbon removal strategies, it still remains contentious whether BECCS is more effective than re/afforestation in terms of atmospheric CO_2_ removal (*2*, *20*, *21*).

Future scenario assessments explore the dynamics of coupled human and Earth systems. On the human system side, there are large inconsistencies in the underlying assumptions for future bioenergy projections across current integrated analysis models used for scenario projections, which may lead to large uncertainties in scenarios (*18*). On the natural system side, while the physical/biogeochemical processes associated with second-generation perennial bioenergy crops (e.g., switchgrass and *Miscanthus*) are important to accurately simulate to what extent bioenergy expansion affects the terrestrial carbon and water dynamics, explicit representation of such key bioenergy crops is largely missing in existing models used for impact assessments. In addition, existing scenario assessments have not yet been performed at high spatial resolutions, at which scales land surface heterogeneity and bioenergy crop-related physical and biogeochemical processes can be appropriately represented and captured.

**Fig. 1.**
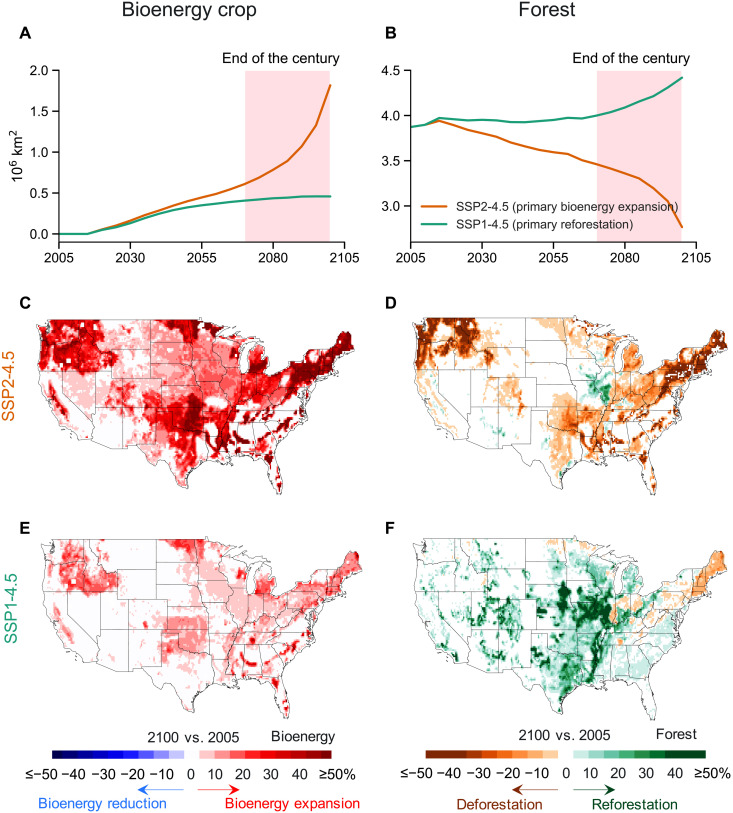
Bioenergy crops are projected to expand rapidly over the CONUS in future social-economic pathways. (**A** and **B**) Plantation areas of bioenergy crops and forest, respectively, over the CONUS from 2005 to 2100 in the SSP2-4.5 and SSP1-4.5 scenarios. (**C** and **E**) Spatial maps of changes in plantation areas of bioenergy crops over the CONUS in SSP2-4.5 and SSP1-4.5, respectively. (**D** and **F**) Spatial maps of changes in plantation areas of forest over the CONUS in SSP2-4.5 and SSP1-4.5, respectively. The spatial value is shown as the difference between 2100 and 2005.

An integrated analysis that jointly considers future bioenergy expansion and forest expansion with consistent underlying assumptions is therefore necessary to quantify the impacts of bioenergy- and forest-driven land use changes on the complex land-carbon-water dynamics. In this study, we develop a multisector and multiscale human-natural system modeling framework, GCAM-Demeter-CLM5_bioenergy_ (see more details in Materials and Methods) (*22*–*24*), which uses consistent assumptions to develop societal pathways and includes explicit biogeophysical and biogeochemical representations of perennial bioenergy crops. We select two co-developed dynamic land use and land cover change (LULCC) scenarios under different socioeconomic development pathways, namely, a primarily bioenergy expansion–based pathway (SSP2-4.5) and a primarily reforestation-based pathway (SSP1-4.5) (see details in Materials and Methods; Fig. 1 and figs. S1 and S2) (*11*, *25*), to assess the viability and potential consequences of bioenergy- and forest-based strategies on carbon removal and water supply. The climate outcome used to drive the simulations is RCP4.5, achieving a radiative forcing of 4.5 W/m^2^ in 2100. We conduct seven numerical experiments over the CONUS at a high resolution (0.125°) to capture fine-scale land surface heterogeneity. These experiments are driven by different combinations of LULCC and climate change (i.e., RCP4.5) scenarios (table S1). Comparison across these experiments allows us to disentangle the relative importance of bioenergy expansion versus reforestation, total LULCC, climate change, and rising CO_2_ on 21st century carbon and water cycle dynamics.

**Fig. 2.**
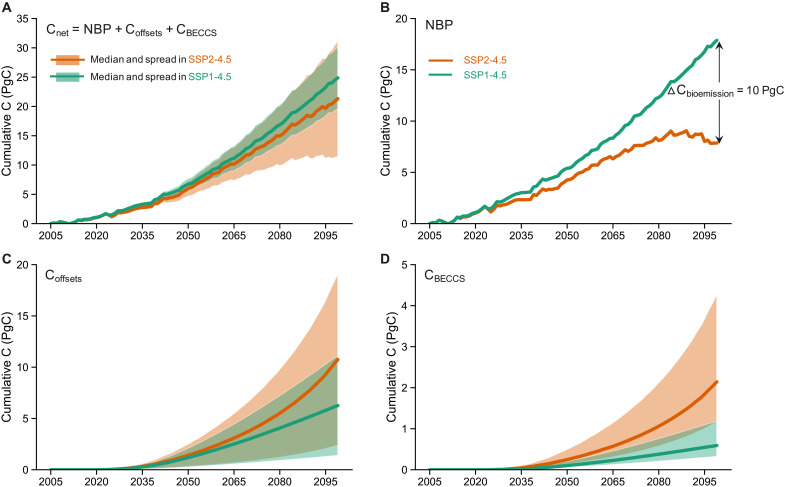
Net land carbon sequestration and its components for the primarily reforestation-based scenario and the primarily bioenergy-based scenario. Cumulative (**A**) net land carbon sequestration [sum of (NBP), carbon fossil fuel offsets due to using bioenergy crops (C_offsets_), and carbon captured via BECCS (C_BECCS_); Eq. 5] and its three components: (**B**) NBP, (**C**) C_offsets_, and (**D**) C_BECCS_ in SSP2-4.5 (orange color, primary bioenergy expansion scenario) and SSP1-4.5 (green color, primary reforestation scenario) during 2005–2100. The upper bound of the spreads in (C) is when bioenergy is offsetting inefficient fossil fuels (i.e., coal), which is a combination of the lowest fossil fuel offset ratio and the highest carbon content of bioenergy crops (Eq. 3; see Materials and Methods for more details). The low bound is the opposite. The upper bound of the spreads in (D) is with the largest percentage of bioenergy used with CCS and the highest CCS capture efficiency (Eq. 4; see Materials and Methods for more details). Opposite is the low end. The upper/lower bound of the spreads in (A) is the sum of the upper/lower bounds in (C) and (D). The ΔC_bioemission_ value (10 PgC) in (B) is carbon emissions due to bioenergy expansion only (Eq. 6).

We find that the net land carbon sequestration is in similar magnitude between the primarily reforestation-based scenario (19.6 to 30.2 PgC; green color spread in Fig. 2A) and the primarily bioenergy-based scenario (11.4 to 31.2 PgC; orange color spread in Fig. 2A). However, the high end of carbon sequestration for the bioenergy-based strategy strongly depends upon assumptions of high future bioenergy crop yields, technology advancement, and CCS effectiveness. Moreover, bioenergy expansion and reforestation (i.e., the full difference between SSP2-4.5 and SSP1-4.5) can cause a larger fraction of the CONUS to experience severe water stress by 2100 due to either reduced water availability or deteriorated water quality. These unintended consequences of bioenergy expansion on carbon emissions and water resources highlight the importance of jointly considering the impacts of replacing existing natural and agro-ecosystems with bioenergy crops on land systems (e.g., carbon, water, and nutrient cycling), technological advancements, and CCS efficiency, as well as the relative benefits and costs of BECCS versus re/afforestation.

## RESULTS

### Impacts of bioenergy expansion and reforestation on carbon uptake

The net land carbon sequestration has similar magnitude between the primarily bioenergy expansion–based scenario (11.4 to 31.2 PgC) and the primarily reforestation-based scenario (19.6 to 30.2 PgC) (Fig. 2A). Here, we define the net land–based carbon sequestration as the cumulative carbon fluxes, including net biome production (NBP; which includes carbon losses from LULCC; Eq. 1 and Fig. 2B), the carbon fossil fuel offsets resulting from using bioenergy crops for energy production (Fig. 2C and Eq. 3), and the carbon captured via BECCS (Fig. 2D and Eq. 4; see Materials and Methods for additional details). These results are consistent with their designed purposes to achieve a similar carbon removal level (i.e., stabilizing global radiative forcing at 4.5 W/m^2^ and limiting warming to 2.4°C or below in 2100).

However, the high-end estimate of the net land carbon uptake in the primarily bioenergy expansion–based scenario largely relies upon several conditions, including assumptions of future improvements in bioenergy crop yields and bioenergy conversion technology as well as implementation of technology to enable efficient CCS (Fig. 2; see Materials and Methods for more details). Without advanced biorefining technology and effective CCS implementation, the net land carbon uptake of the primary bioenergy expansion scenario would result in about 70% weaker carbon sequestration benefits compared to those predicted for the primary reforestation scenario (Fig. 2A). Note that the primary reforestation scenario also involves a certain degree of bioenergy expansion (0.46 million km^2^; Fig. 1A), leading to carbon uptake benefits in terms of carbon fossil fuel offsets and CCS capture. In particular, the maximum possible value of carbon fossil fuel offsets in the primary reforestation scenario (SSP1-4.5) is equivalent to the average value in the primary bioenergy expansion scenario (SSP2-4.5) (Fig. 2C). In addition, the primary net C benefits in both scenarios may come more from the carbon fossil fuel offsets than from the CCS (Fig. 2).

**Fig. 3.**
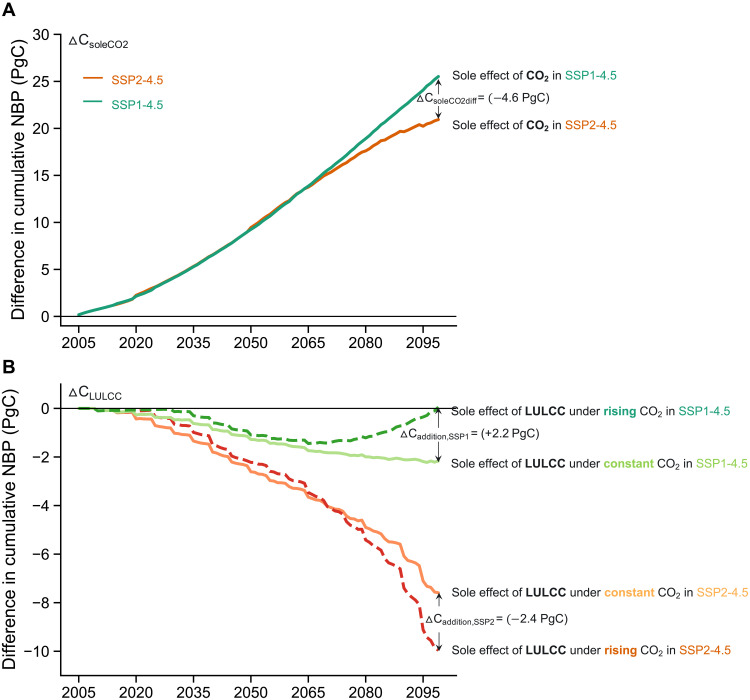
The CO_2_ fertilization effect and the additional carbon sink capacity in the primary bioenergy expansion scenario and the primary reforestation scenario. Differences in cumulative NBP attributed to (**A**) CO_2_ fertilization and (**B**) LULCC in SSP2-4.5 (red colors, primary bioenergy expansion) and SSP1-4.5 (green colors, primary reforestation) scenarios. ﻿﻿The sole effect of CO_2_ fertilization (ΔC_soleCO2_) is estimated as the difference in NBP between two scenarios with the same LULCC but constant or rising CO_2_ (Eqs. 7 and 8). Similarly, the sole effect of LULCC (ΔC_LULCC_) is estimated as the difference in NBP between two scenarios with the same climate (RCP4.5) but different LULCC. The LULCC effect is further separated depending on whether the two scenarios are driving by constant (Eqs. 10 and 13) or rising CO_2_ (Eqs. 11 and 14). The ΔC_soleCO2diff_ value (−4.6 PgC; Eq. 9) in (A) is the difference of carbon uptake in response to rising CO_2_ between SSP2-4.5 and SSP1-4.5. The ΔC_addition,SSP2_ (−2.4 PgC; Eq. 12) and ΔC_addition,SSP1_ (+2.2 PgC; Eq. 15) values in (B) are changes in the additional carbon sink capacity in SSP2-4.5 and SSP1-4.5, respectively.

We find that changes in NBP (Fig. 2B), a major portion of the net land carbon uptake, is dominated by the CO_2_ fertilization effect (i.e., enhanced natural carbon uptake due to elevated CO_2_ that can stimulate photosynthesis rates) (*26*–*28*), rather than climate change and LULCC (fig. S6C). However, the CO_2_ fertilization effect is 4.6 PgC weaker in the primary bioenergy expansion scenario compared to the primary reforestation scenario (Fig. 3A and Eq. 9) due to a lower sink capacity associated with the smaller forested area in the primary bioenergy expansion scenario. The deforestation in the bioenergy-based SSP2-4.5 scenario results in a 2.4 PgC weaker forest carbon sink (Fig. 3B and Eq. 12), whereas the reforestation in SSP1-4.5 supports the drawdown of an additional 2.2 PgC sink capacity by 2100 (Fig. 3B and Eq. 15; see more details in Materials and Methods) (*29*). Moreover, land use conversions for bioenergy crop plantation (*1*, *2*) (figs. S1 and S2) drive 10 PgC of reduced carbon uptake in the natural vegetation by 2100 in SSP2-4.5 (Fig. 2B and Eq. 6; see Materials and Methods for more details). The impact of bioenergy expansion on terrestrial carbon stocks depends on the counterfactual used. Our results suggest that, compared to pathways with forest expansion (*30*), bioenergy deployment has a more adverse effect on NBP than existing land use (Figs. 2 and 3).

The positive impacts of the primary forest-based strategy over the primary bioenergy-based strategy on NBP are more substantial during the late 21st century (Fig. 2B) when most forests and bioenergy crops are planted (Fig. 1, A and B). In detail, forest area in SSP1-4.5 increases by 0.1 and 0.4 million km^2^ during 2005–2069 and 2070–2099, respectively (Fig. 1A), and bioenergy area in SSP2-4.5 increases by 0.5 and 1.2 million km^2^ during 2005–2069 and 2070–2099, respectively (Fig. 1B). As a result, in SSP2-4.5, the cumulative NBP starts to decline during 2070–2099 (Fig. 2B) when it is experiencing the highest degree of bioenergy expansion. In contrast, carbon starts accumulating on land more quickly during 2070–2099 in the primary reforestation scenario SSP1-4.5 (Fig. 2B). The forests that are mainly planted in the late 21st century (Fig. 1B) are accumulating and will likely continue to accumulate carbon in this primary reforestation scenario (Fig. 2B).

### Impacts of bioenergy expansion on water availability and quality

**Fig. 4.**
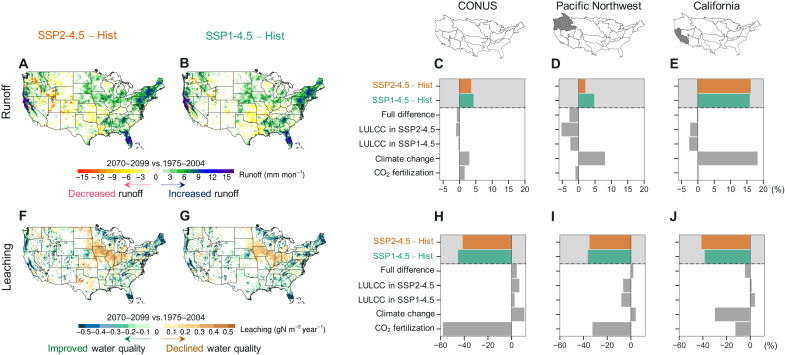
Changes in climate and land uses result in large differences in runoff and nitrogen leaching over the CONUS by the end of the century. Changes are shown as the absolute differences between the end of the century (2070–2099) relative to the historical period (1975–2004) for the SSP2-4.5 (first column) and SSP1-4.5 (second column) scenarios. Changes in runoff (**A** to **E**, first row) and nitrogen leaching (**F** and **G**, second row) for (C and **H**) CONUS, (D and **I**) Pacific Northwest region (high bioenergy expansion), and (E and **J**) California region (low bioenergy expansion) in the two scenarios and attributions to individual components including full difference between SSP2-4.5 and SSP1-4.5, LULCCs in SSP2-4.5 and SSP1-4.5, climate change, and CO_2_ fertilization (see Materials and Methods for more details).

Both future water availability and water quality are projected to decline due to bioenergy expansion. The decrease in runoff associated with bioenergy expansion and reforestation (i.e., the full difference between SSP2-4.5 and SSP1-4.5) (−0.7%; Fig. 4, A, B, and C, and table S2) is mainly driven by higher evapotranspiration (without the application of irrigation) of bioenergy crops due to their longer growing seasons compared to the replaced land covers (fig. S8) (*24*). However, the positive impacts of increased precipitation (+2.9%) and elevated CO_2_ (+1.5%) (*31*) on runoff offset the opposing effect of bioenergy expansion, leading to an overall increase in CONUS-wide runoff by 2100 in the primary bioenergy expansion scenario (+3.5%; Fig. 4C). At the basin scale, however, changes in runoff are highly variable across regions, especially in basins with similar climate but distinct bioenergy expansion extents (fig. S5). For example, decreased runoff due to bioenergy expansion is seen in the intensive bioenergy Pacific Northwest region (−2.7%; Fig. 4D) that accounts for 10% of total U.S. bioenergy crop plantations (0.18 million km^2^ by 2100; fig. S5), but is not notable in the California region (−0.2%; Fig. 4E) that has only 1.1% of total bioenergy expansion (0.02 million km^2^ by 2100; fig. S5).

The increase in nitrogen leaching (+4.1%) due to bioenergy expansion and reforestation (i.e., the full difference between SSP2-4.5 and SSP1-4.5) (Fig. 4, F, G, and H, and table S2) comes from the application of modest amounts of fertilizer for switchgrass (*24*). The decreased CONUS-average nitrogen leaching by 2100 in the primary bioenergy expansion scenario (−41.0%; Fig. 4H), which consists of changes in climate, CO_2_, and LULCC, is attributed to the dominant positive impact of CO_2_ fertilization on improving nitrogen uptake efficiency (*32*). This CO_2_ fertilization effect decreases nitrogen leaching by −57.8% (Fig. 4H). Regionally, however, we observe a bimodal spatial distribution of both degraded (central United States due to fertilization in agriculture) and improved (the regions outside central United States) water quality (Fig. 4F). In contrast, reforestation does not require fertilizer application and therefore does not drive increases in nitrogen leaching (2.2%) in the primary reforestation scenario (Fig. 4, G and H).

### Projected water stress over the CONUS

**Fig. 5.**
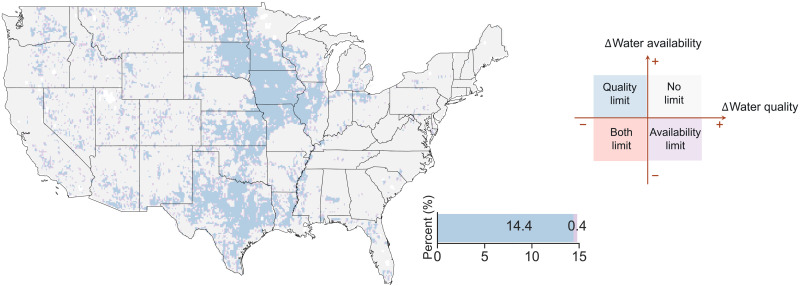
The CONUS land areas that are projected to experience severe water stress caused by bioenergy expansion and reforestation (i.e., the full difference between SSP2-4.5 and SSP1-4.5) by the end of the century. Estimates were made by comparing the difference in water availability/quality between the end of the century (2070–2099) relative to the historical period (1975–2004). Water stress classifications (i.e., no limit, availability limit, quality limit, and both limit) can be found in Materials and Methods (Eqs. 18 to 21).

We find that bioenergy expansion could have unintended consequences in exacerbating water stress by reducing either water availability or water quality. At the annual time scale, bioenergy expansion and reforestation (i.e., the full difference between SSP2-4.5 and SSP1-4.5) can lead to one-seventh (14.8%) of CONUS area to experience severe water stress (see Materials and Methods for further details) by the end of the century compared to the historical period (Fig. 5).

Other aspects of climate change interact with bioenergy expansion to contribute to even larger areas of water stress. In the primary bioenergy expansion scenario, we find that nearly one-fourth of the land area (24.6%) over the CONUS will suffer severe water stress by the end of the century (fig. S9A), mainly driven by bioenergy expansion and climate change (Fig. 4, C and H). The contradictory effects of bioenergy expansion, climate change, and CO_2_ fertilization on water availability and quality (Fig. 5) result in a highly uneven distribution of water stress changes in SSP2-4.5 (fig. S9). Areas under quality-induced water stress are mainly distributed in the Midwest, which are covered by a large fraction of fertilized croplands for bioenergy crops (fig. S2A) and corn (fig. S2F), implying potentially heightened risk of water stress due to worsened water quality, although shortage in water availability alone is not considerable (*33*). Most availability-limited areas are in the western United States due to decreased precipitation (fig. S4) and increased evapotranspiration (fig. S8). Compared to the primary bioenergy expansion scenario, the primary reforestation scenario results in slightly less area exhibiting severe annual water stress (+20.2%) (fig. S9). In particular, there is less area experiencing quality-limited water stress in SSP1-4.5, because reforestation does not require fertilizer application and therefore does not drive any decreases in water quality (Fig. 4H). Note that, as the primary reforestation scenario involves almost a third of the bioenergy expansion in the primary bioenergy expansion scenario, the impact of bioenergy expansion and reforestation (i.e., the full difference between SSP2-4.5 and SSP1-4.5) on severe water stress (14.8%) is greater than the case if these two scenarios are directly compared (24.6% versus 20.2%).

With projected U.S. population of 525 million in SSP2-4.5 by the end of the century (*34*, *35*), our results imply that 130 million people (see Materials and Methods for more details, Eq. 22) may suffer severe water stress due to either reduced water availability or worsened water quality in this primary bioenergy expansion scenario, while bioenergy expansion and reforestation (i.e., the full difference between SSP2-4.5 and SSP1-4.5) can potentially cause 40 million U.S. people to experience severe water limitation by 2100. These findings highlight the potential negative consequences of bioenergy expansion on sustainable water supply.

## DISCUSSION

In this study, we develop a human-natural system modeling framework across various sectors (e.g., socioeconomic, land, water, and carbon) and scales (e.g., water basin and grid) to quantify the competing effects of bioenergy- versus reforestation-based strategies on carbon uptake and water supply. To our knowledge, this is the first study to explicitly investigate the role of 21st century second-generation perennial bioenergy crop expansion on carbon uptake and water stress (both quantity and quality) concurrently. We find that the net land carbon uptake in the bioenergy expansion–focused scenario can range from 11.4 to 31.2 PgC by 2100. The high-end estimate of the net carbon sequestration benefit largely depends on rising bioenergy crop yields, technology advancements, and BECCS effectiveness. At the low end, the net land carbon sequestration in the primary bioenergy expansion scenario is ~70% lower than that in the primary reforestation scenario. Our analysis of land carbon uptake also reveals a few adverse effects of bioenergy-based strategies compared to forest-based strategies. For instance, ~10 PgC is emitted due to land use conversions that are required to clear land for sufficient bioenergy production. In addition, there is a decreased carbon uptake in response to rising CO_2_ (−4.6 PgC) in the bioenergy-based scenario compared to the reforestation-based scenario. Moreover, the bioenergy-focused scenario results in a larger fraction of the CONUS area facing an increase in water stress due to the combined impacts of water quantity reduction and water quality degradation compared to the reforestation-focused scenario. Our results imply that approximately 130 million people will live in regions with water limitation by the end of the century in the primary bioenergy expansion scenario, with about one-third of that population (40 million people) being associated directly with the impact of bioenergy expansion and reforestation. The fact that a larger area would suffer water stress with a larger increase in bioenergy plantation due to degrading water quality is consistent with previous studies, which find exacerbated eutrophication as an unintended consequence of bioenergy expansion (*18*, *36*). The carbon release associated with land conversion from natural vegetation to biomass plantations and the regional hot spots that suffer either availability- or quality-constrained water stress highlight the importance of assessing impacts of bioenergy expansion in a manner that jointly considers the impacts on carbon uptake and water conditions. Such side effects could also emerge in other parts of the world with similar or even more aggressive bioenergy crop expansion policies and could further increase the risk of water stress over regions that are already susceptible to climate change. For instance, there is mounting evidence that Asia will experience increased risk of eutrophication (*18*, *36*) under a warming climate. With anticipated expansion of bioenergy crops in this region, which would reach 3.9 million km^2^ by 2100 in SSP2-4.5 (*23*), more than twice as much as that projected in the United States, the impact of eutrophication could be even worse.

There are some key features of this study that are worth mentioning. First, the primary bioenergy expansion and the primary reforestation scenarios are co-developed by the same coupled human-Earth system model, and therefore, their underlying structural assumptions are self-consistent. We use the same model for both scenarios to minimize the uncertainties in land use projections due to differences in assumptions across models (*18*). In addition, coupled human-Earth system models used in most existing studies are often configured in such a way that divides the entire world into a limited number of regions (*37*). In this study, we use Demeter-downscaled land uses, which are in a much finer grid-based resolution and more suitable for Earth system model simulations. At this fine spatial scale, subregional land surface heterogeneity (e.g., spatial variability of agricultural operations for bioenergy crops) is more adequately captured. Moreover, although our study focuses on the United States, the multisector and multiscale modeling framework developed in this study is highly flexible and can be generalized to other regions or applied at the global scale.

This study also reveals some challenges that deserve further consideration. First, the dynamic LULCC experiments (i.e., SSP2-4.5 and SSP1-4.5) examined in this study only represent two prototype socioeconomic scenarios for feasible future societal development and associated land uses. Many other socioeconomic and land use scenarios, even at the same RCP level, could result in similar carbon removal levels (*25*). Nevertheless, the examined pathways in this study represent high- and low-end bioenergy expansion scenarios, which can shed light on the potential lower and upper bounds of the impacts of large-scale bioenergy crop plantations. In addition, while fertilizer application is an important driver of changes in water quality, only total fertilizer application rates are provided for each crop without details about how and when that fertilizer is applied (*38*, *39*). The timing and frequency of seasonal application of fertilizer (*40*), which is currently treated relatively simplistically in Community Land Model (CLM) as a steady application over a set number of days at the beginning of the growing season, is likely to affect leaching rates. Moreover, this study relies on a single set of ﻿meteorological forcing to evaluate the impact of climate change and LULCC on carbon emissions and water conditions, while previous studies (*18*, *41*) have demonstrated that the impacts of LULCC could be sensitive to the nature and magnitude of projected climate change. Therefore, multimodel ensemble-based analysis considering other SSP-RCPs and coupled human-Earth system models should be conducted in the future to systematically quantify the impacts and associated uncertainties of climate change and bioenergy expansion on the complex energy-land-carbon-water dynamics. Meanwhile, this study starts future projections in 2005, as this time period is consistent with the land use data (*11*). Future analysis could use a more updated dataset with a more up-to-date study period. The climate forcing used in this study is RCP4.5, as lower RCP simulations at a high resolution (0.125°) from the Regional Earth System Model (RESM) were not available. Future study could be performed for lower radiative targets that are compatible with 1.5° or 2°C warming once the forcing data become available. Note also that there are two-way feedbacks between human and natural systems. For instance, in reality, LULCC can alter climate by altering biophysical properties and fluxes from the land surface (e.g., surface albedo and sensible and latent heat) (*2*), which could then alter decisions on future land use and land use conversions. Land-based responses can also potentially interact with other sectors (e.g., energy and economy) within the human system through anthropogenic activities such as adaptation (*42*). However, this study only considers the one-way impact of changes in climate and land use on terrestrial land surface processes without feedback to/from the atmosphere or the human system. To understand the coevolution of human and Earth systems requires a fully coupled modeling framework that can examine the complex interactions, multidirectional feedbacks, and uncertainties between human and natural systems simultaneously (*43*, *44*). Despite the uncertainties described here, this study nonetheless points to the need for more holistic analyses of bioenergy-based carbon removal solutions that consider all dimensions of the complex human and natural systems as well as the unintended consequences (e.g., bioenergy expansion–associated carbon emissions and heightened water stress) and trade-offs (e.g., cost of choosing bioenergy production over reforestation) of large-scale bioenergy plantations.

## MATERIALS AND METHODS

### Overall modeling framework

We develop a multiscale and multisector modeling framework, namely, GCAM-Demeter-CLM5_bioenergy_, to examine the consequences of different societal pathways (e.g., primary bioenergy expansion and primary reforestation) on carbon cycle and water conditions. GCAM (Global Change Analysis Model) (*22*) is used to explore the dynamics of the coupled human-Earth system and the response of this system to global changes. Demeter is a LULCC disaggregation model (*23*, *37*, *45*) used to downscale continental and global scale land use and land cover to various grid-based spatial resolutions. CLM5_bioenergy_ is the version 5 of the CLM with explicit representation of perennial bioenergy crops (*24*, *38*). The GCAM-Demeter-CLM5_bioenergy_ modeling framework can capture the dynamic interactions among energy, water, economy, land, climate, and other sectors. RCP4.5 is considered in this study (i.e., achieving a radiative forcing of 4.5 W m^−2^ in 2100). Two combinations of SSPs and climate outcome (RCP4.5) from GCAM, namely, SSP2-4.5 and SSP1-4.5, are selected to represent bioenergy expansion–dominated and re/afforestation-dominated scenarios, respectively. These two scenarios are downscaled from GCAM projections by Demeter to provide 0.05° land use maps over the CONUS every 5 years during 2005–2100 that can be used by CLM5_bioenergy_.

### Future LULCC projections

We extract downscaled land use scenarios for SSP1-4.5 (reforestation based) and SSP2-4.5 (bioenergy based) over the CONUS during 2005–2100 from a global dataset produced by GCAM and downscaled by Demeter (*11*). The dataset contains downscaled land use projections at a 0.05° resolution under multiple combinations of SSPs and RCPs (*11*). We aggregate the 0.05° land use projections to a 0.125° resolution to conduct the simulations. Here, we summarize key land use changes relevant to this study. For a full overview of the land use projections and the downscaling details, interested readers are referred to (*11*).

GCAM was driven by climate output from five global climate models (GCMs) (i.e., IPSL, GFDL, HADGEM, MIROC, and NORESM) to generate a range of land use scenarios chosen by the Scenario Model Intercomparison Project (ScenarioMIP) for ﻿the Coupled Model Intercomparison Project Phase 6 (CMIP6) experiments based on human decisions (*11*, *46*). SSPs are designed to describe plausible alternative changes in various aspects of society such as demographic, economic, technological, social, governance, and environmental factors. They are intended as a description of plausible future conditions at the level of large regions that can serve as a basis for integrated scenarios of emissions and land use as well as climate impacts, adaptation, and vulnerability analyses. The SSPs as generated by GCAM include changes in all of those factors. However, because this study focuses on studying the impact of large-scale bioenergy expansion from the resulting set of land use scenarios, we selected the scenarios with the largest bioenergy expansion for SSP2-4.5 and the smallest bioenergy expansion for SSP1-4.5, which were simulations driven by climate output from the MIROC model. Note that there is actually not much difference in the trends and magnitudes of bioenergy plantation areas within the United States among the land use results driven by the five GCM inputs (*11*).

The latest version of GCAM divides the global land area into 384 regions based on 32 geopolitical regions and 235 water basins (22 region-basin combinations for the United States) (*11*, *22*). The basin size typically ranges from a few hundreds to millions of square kilometers. This scale of information is not directly suitable for use in CLM5, which is a gridded model. Therefore, spatial downscaling of water basin level land use is critical to link GCAM and CLM5 to investigate the effects of LULCC on ecosystem functions and to better represent the interactions between human and natural systems. For continental and global scale land use downscaling, Demeter (*23*, *37*, *45*) has been parameterized and applied.

GCAM includes a high-yield perennial bioenergy crop in SSP2-4.5 (e.g., switchgrass). By 2100, approximately 2 million km^2^ are projected for bioenergy crop plantations in SSP2-4.5 over the CONUS by replacing forest, shrub, grass, and food crop areas. Compared to 2005, the forest, shrub, grass, and food crop areas in 2100 decrease by 1.11, 0.21, 0.63, and 0.06 million km^2^, respectively, to accommodate the increased bioenergy crop area (figs. S1 and S2). SSP1-4.5 has substantial land use change due to increased forest cover (0.55 million km^2^; Fig. 1A) (*7*, *25*) and a certain degree of bioenergy expansion (0.46 million km^2^; Fig. 1A). The reforestation and bioenergy expansion in this scenario are achieved by replacing shrub, grass, and food crop areas. Specifically, the reductions in area of these three land cover types between 2100 and 2005 are 0.16, 0.19, and 0.7 million km^2^, respectively (figs. S1 and S2).

### Historical and future climate scenarios

The regional simulations conducted in this study are executed over 1975–2100 driven by historical (1975–2004) and future (2005–2100) projected climate forcing under the RCP4.5 climate scenario. Hourly meteorological forcing is the product from the RESM at a 0.125° resolution (figs. S3 and S4). Compared to the historical period, the mean annual precipitation and temperature in RCP4.5 increase by 25.4 mm and 2.1 K, respectively, by the end of the century. Spatially, most of the regions over the CONUS experience wet trends, except for some small areas in the mid-south United States (e.g., Arkansas-White-Red and Texas-Gulf regions; fig. S3A). The entire United States consistently experiences a warmer climate, while the central United States is more notable (figs. S3 and S4). An earlier study (*33*) has evaluated RESM historical forcing using observed temperature and precipitation and found that it can reasonably capture the observed seasonal temperature and precipitation and extreme precipitation (*45*). Future atmospheric CO_2_ concentration is prescribed using the SSP2-4.5 data from the Community Earth System Model (CESM) (fig. S4E).

### CLM5 with explicit representation of perennial bioenergy crops

CLM5 represents land cover heterogeneity and ecosystem structure of the land surface and consists of components or submodules related to biogeophysics, biogeochemistry, hydrology, human dimensions, and ecosystem dynamics (*38*, *47*). CLM5 is capable of simultaneously simulating the energy, water, carbon, and nitrogen cycle dynamics driven by climate and ﻿land use changes. To represent land surface heterogeneity, CLM5 divides each grid cell into multiple land units, each of which consists of multiple snow/soil columns that are occupied with different plant functional types (PFTs).

For carbon-nitrogen cycling, CLM5 prognostically simulates carbon and nitrogen state variables for vegetation, including leaf, live stem, dead stem, live coarse root, dead coarse root, fine root, and grain pools. In addition to the vegetation C and N pools, CLM5 includes a series of decomposing C and N pools as vegetation successively breaks down to coarse wood debris, and/or litter, and subsequently to soil organic matter. These prognostic variables are used by the biophysical module to simulate water and energy budget terms.

For simulating the CO_2_ fertilization effect in CLM5, time-varying CO_2_ concentration is coupled to biogeochemical cycles in CLM5, with photosynthetic capacity and stomatal resistance directly responding to changes in the CO_2_ concentration. The photosynthetic capacity of natural vegetation is parameterized by the Leaf Utilization of Nitrogen for Assimilation (LUNA) module (*48*), as a function of CO_2_ concentration, temperature, radiation, relative humidity, and day length (*49*). The photosynthetic capacity of crops is estimated on the basis of the maximum rate of carboxylation, leaf nitrogen content, and day length. Meanwhile, photosynthetic capability determined by these environmental factors can modulate stomatal resistance. Therefore, the increased CO_2_ concentration can regulate physiological (e.g., stomatal opening and closure) and phenological (e.g., leaf area index) responses in CLM5, which, in turn, can affect the terrestrial carbon and water cycle dynamics. The representation of CO_2_ fertilization in current generation land models remains highly divergent (*50*). CLM5 simulations show better agreement with observed responses to experimental N and CO_2_ enrichment than previous versions of the model (*51*), although Fisher *et al*. (*49*) point out that CO_2_ fertilization rates in CLM5 are strongly dependent on relatively unconstrained parameter values. In addition, the CLM5 representation of mortality through fire, drought, or other events is relatively simplistic. Nonetheless, CLM5 is among the best performing current generation models (*52*). Results presented here, therefore, should be considered with these model structural and parametric uncertainties taken into account.

In the hydrology module, CLM5 simulates hydrologic processes including interception, throughfall, canopy drip, snow accumulation and melt, water transfer between snow layers, infiltration, evaporation, surface runoff, subsurface runoff, redistribution within the soil column, and groundwater discharge and recharge. The complete surface and subsurface hydrologic processes represented in CLM5 are shown in fig. S7.

CLM5 can simulate transient LULCCs through a prescribed landuse.timeseries dataset (*47*). Changes in areas of PFTs are performed annually on 1 January of the year. Fertilizer and irrigation (*53*) are applied only to crop PFTs and are applied depending on rules from the crop module. Fertilizer application rates and irrigation areas are also set annually. Feralization and irrigation affect the water quality and water availability, as fertilizer is an input for nitrogen leaching and irrigation could alter energy fluxes. For a full overview of CLM5 and the process of leaching, interested readers are referred to (*38*, *47*).

Compared to annual crops, perennial bioenergy crops have longer growing seasons, greater leaf areas, and higher productivity, which can lead to larger carbon uptake, higher transpiration, and lower runoff. Cheng *et al*. (*24*) have implemented two perennial bioenergy crops, namely, switchgrass and *Miscanthus*, into CLM5 by modifying parameters associated with photosynthesis, phenology, allocation, decomposition, and carbon cost of nitrogen uptake. At the harvest time, 70% of bioenergy crops’ aboveground biomass, including live stem and leaf biomass, is removed and put into a 1-year harvested bioenergy product pool to represent the harvest of lignocellulosic bioenergy crops (*24*). The remaining 30% carbon is moved to the litter carbon pools. The carbon in the 1-year bioenergy product pool is released back to the atmosphere over 1 year (C_bioenergy_prodloss,1-year_ in Eq. 2) in CLM5, as bioenergy production in the United States is targeted to match demand within a year. The concomitant land management practices for perennial bioenergy crops are also integrated, including little fertilizer (e.g., 56 kg/ha per year) for switchgrass, no fertilizer for *Miscanthus*, and no irrigation for either perennial crop during the growing season. For annual food crops, the fertilizer applied for corn and soybean in the United States is 150 and 17 kg/ha per year, respectively. Irrigation is applied for food crops in areas equipped for irrigation, as defined by Demeter (*11*), if the available soil water is below a specified soil moisture target (*54*).

### Simulations

In this study, we run CLM5 over the CONUS (67°W to 125°W, 25°N to 53°N, 464 × 224 grid cells) at a 0.125° spatial resolution with a 30-min time step, driven by the RESM climate forcing data (see the “Historical and future climate scenarios” section). Seven numerical experiments are conducted, driven by different combinations of LULCC and climate change scenarios (table S1). Land cover information (i.e., the percentage of each vegetation type) used in the historical, climate change, and climate + CO_2_ simulations (table S1) is derived from the Moderate Resolution Imaging Spectroradiometer (MODIS) satellite data using the methods proposed in (*55*), corresponding to the year 2000 condition. Future dynamic LULCC simulations (SSP2-4.5 and SSP1-4.5) from 2005 to 2100 use transient spatial land use time series that are projected by GCAM and downscaled by Demeter (see the “Future LULCC projections” section and figs. S1 and S2). The 30-year historical forcing data are recycled for 800 years to spin up the model for the carbon and nitrogen pools to reach equilibrium. The performance of CLM5 in simulating land surface fluxes over the CONUS from 1979 to 2018 has been evaluated against various in situ and remote sensing datasets in an earlier study (*56*). Two regions (i.e., Pacific Northwest and California) are selected for further analyses due to their similar climate but distinct magnitudes of bioenergy expansion (fig. S5). Specifically, the changes in mean annual precipitation and temperature between the end of the century and the historical period are 67/63 mm and 1.9/2.0 K for the Pacific Northwest/California regions. The increases in plantation areas of bioenergy crops by 2100 are 0.18 and 0.02 million km^2^ for the two regions, respectively.

### Net biome production

NBP (Fig. 2B) includes net carbon sequestration by ecosystems and carbon losses due to fire, harvest mortality, and land use, with positive/negative values indicating carbon sequestration/release on land. Mathematically, NBP can be calculated as

$$\text{NBP}=\text{NEP}–C_{\text{fireloss}}–C_{\text{landuse}}=\text{GPP}–\text{ER}–C_{\text{fireloss}}–C_{\text{landuse}}$$ (1)

where NEP is the net ecosystem production, GPP is the gross primary production, ER is the ecosystem respiration, and C_fireloss_ and C_landuse_ are the carbon emissions associated with fire loss and land use change, respectively. Positive values of C_fireloss_ and C_landuse_ indicate carbon loss to the atmosphere.

C_landuse_ is the total C that is directly emitted from land cover conversion (C_conversion_) as well as from the grain and wood product pools, including the 1-year harvested bioenergy product pool (C_bioenergy_prodloss,1-year_), the 1-year grain to food product pool (C_grain_loss,1-year_), and the 10-year (C_woodprodc_loss,10-year_) and 100-year (C_woodprodc_loss,100-year_) wood product pools, as calculated in Eq. 2 below. Here, the 1-, 10-, and 100-year product pools mean that these fluxes would exponentially decay and loss to the atmosphere over these certain time periods

$$\begin{array}{c} C_{\text{landuse}}=C_{\text{conversion}}+C_{\text{bioenergy}\_\text{prodloss},1-\text{year}}+C_{\text{grain}\_\text{loss},1-\text{year}}+\\ C_{\text{woodprodc}\_\text{loss},10-\text{year}}+C_{\text{woodprodc}\_\text{loss},100-\text{year}} \end{array}$$ (2)

### Carbon fossil fuel offsets due to using bioenergy crops calculation

The additional advantage of using bioenergy crops is that any energy produced with bioenergy means a reduction in the need for fossil fuels (*57*, *58*). In other words, the amount of bioenergy used to produce final energy prevents carbon being emitted to the atmosphere from fossil fuel burning and thereby this carbon offset can be considered an “effective carbon sink.” Therefore, this carbon fossil fuel offset should be added to the overall land carbon uptake to account for the full land-based carbon sequestration.

To calculate the carbon fossil fuel offsets, there are a few relevant factors to consider. The first is how much final energy (e.g., electricity or liquid fuels) is produced with a certain amount of bioenergy. The second is how much fossil fuels are needed to produce the same amount of final energy as from bioenergy. The third is the carbon content of the bioenergy and the fossil fuel (offsetting 1 unit of natural gas will be less emissions reduction than 1 unit of coal). The fourth is whether bioenergy is actually displacing fossil fuels. For the fourth point, we assume that bioenergy offsets fossil fuels in this study, as it is unlikely that bioenergy is replacing nonfossil fuels in scenarios with a low radiative forcing. The first and second points are captured by a factor called *R*_offset_ as explained below. Therefore, the carbon fossil fuel offsets (C_offsets_; Fig. 2C), which is the amount of carbon not emitted to the atmosphere as a result of using energy from biomass rather than fossil fuels, can be calculated as follows

$$C_{\text{offsets}}=\frac{C_{\text{bioenergy}\_\text{prodloss},1-\text{year}}}{R_{\text{offset}}}*{\text{CC}}_{\text{fossil}}/{\text{CC}}_{\text{bioenergy}}$$ (3)

where C_bioenergy_prodloss,1-year_ is the carbon released to the atmosphere from the 1-year harvested bioenergy product pool. *R*_offset_ is the fossil fuel offset ratio, ranging from 0.5 (meaning 0.5 EJ/year of bioenergy is needed to offset 1 EJ/year of fossil fuels) to 2 (meaning 2 EJ/year of bioenergy is needed to offset 1 EJ/year of fossil fuels) in both SSP2-4.5 and SSP1-4.5 in GCAM. The low end happens with efficient bioenergy and inefficient fossil fuels. The high end is the opposite. Higher *R*_offset_ implies that bioenergy crops are inefficient; thereby, a unit of bioenergy crop will produce less overall energy and offset fewer fossil fuels. Therefore, C_offsets_ is in a reverse relationship with *R*_offset_. CC_bioenergy_ is the carbon content of bioenergy crops, which is ﻿the amount of carbon emitted per unit of energy for bioenergy. The value of CC_bioenergy_ is 23 kg C/GJ in GCAM. CC_fossil_ is the carbon content of fossil fuels. The values of CC_fossil_ are 14.2 kg C/GJ for natural gas, 19.6 kg C/GJ for oil, and 27 kg C/GJ for coal in GCAM. The upper bound of the spreads in Fig. 2C is when bioenergy is offsetting inefficient fossil fuels (i.e., coal), which is a combination of the lowest *R*_offset_ (i.e., efficient bioenergy but inefficient fossil fuels such as coal) and the highest CC_fossil_ (i.e., bioenergy is offsetting coal). The lower bound is the opposite.

### Carbon uptake via BECCS calculation

The carbon uptake via BECCS (C_BECCS_; Fig. 2D) is calculated as

$$C_{\text{BECCS}}=C_{\text{bioenergy}\_\text{prodloss},1-\text{year}}*{\text{frac}}_{\text{CCS}}*{\text{Eff}}_{\text{CCS}}$$ (4)

where frac_CCS_ is the percentage of bioenergy crops used with CCS. Eff_CCS_ is the CCS capture efficiency. In GCAM, frac_CCS_ is assumed to be 60 and 30% in SSP2-4.5 and SSP1-4.5, respectively. Eff_CCS_ is assumed to range from 85 to 95% in electricity depending on the year, and from 26 to 91% in liquid fuels depending on the conversion pathway and capture rate. Here, the range for Eff_CCS_ is set to be 26 to 95%. The upper bound of the spreads in Fig. 2D is corresponding to the largest frac_CCS_ and the highest Eff_CCS_. Opposite is the lower end.

### Net land carbon uptake calculation

The net carbon sequestration benefit (C_net_; Fig. 2A) includes NBP, C_offsets_, and C_BECCS_

$$C_{\text{net}}=\text{NBP}+C_{\text{offsets}}+C_{\text{BECCS}}$$ (5)

### Carbon emissions due to bioenergy expansion calculation

The cumulative carbon released due to land use conversions for bioenergy crop plantation is calculated as the difference in cumulative NBP between the primary reforestation scenario and the primary bioenergy expansion scenario (Eq. 6 and Fig. 2B), as the primary reforestation scenario represents the opposite situation (i.e., primary reforestation rather than primary bioenergy expansion)

$$\Delta C_{\text{bioemission}}=\sum {\text{NBP}}_{\text{SSP}1-4.5,\text{year}2100}–\sum {\text{NBP}}_{\text{SSP}2-4.5,\text{year}2100}$$ (6)

where ∑NBP_SSP1 − 4.5_ and ∑NBP_SSP2 − 4.5_ are the cumulative NBP in 2100 in SSP1-4.5 and SSP2-4.5, respectively.

### Attribution analysis of bioenergy expansion, LULCC, climate change, and CO_2_ fertilization

In this study, we examine how changes in carbon and water budgets are driven by the following five key factors, including (i) bioenergy expansion and reforestation (i.e., the full difference between SSP2-4.5 and SSP1-4.5), (ii) total LULCC in SSP2-4.5, (iii) total LULCC in SSP1-4.5, (iv) climate change, and (v) CO_2_ fertilization. The contribution of each individual factor can be quantified through the following pairwise simulation comparisons: (i) SSP2-4.5 versus SSP1-4.5, (ii) SSP2-4.5 versus RCP4.5 + CO_2_, (iii) SSP1-4.5 versus RCP4.5 + CO_2_, (iv) RCP4.5 versus historical run, and (v) RCP4.5 + CO_2_ versus RCP4.5 (Fig. 4 and table S2).

### Difference of carbon uptake in response to rising CO_2_

We estimate the sole effect of CO_2_ fertilization (ΔC_soleCO2_; Fig. 3A) on carbon uptake as the difference in NBP between two scenarios with the same LULCC but constant or rising CO_2_ as given in Eqs. 7 and 8 below. The difference of carbon uptake in response to rising CO_2_ between SSP2-4.5 and SSP1-4.5 by 2100 (ΔC_soleCO2diff_; −4.6 PgC in Fig. 3A) is calculated as the difference between ΔC_soleCO2,SSP2_ and ΔC_soleCO2,SSP1_ in 2100 as shown in Eq. 9. Please refer the scenario names in the subscript (e.g., SSP2-4.5[consCO2]) to table S1.

$$\Delta C_{\text{soleCO}2,\text{SSP}2}=\sum {\text{NBP}}_{\text{SSP}2-4.5}–\sum {\text{NBP}}_{\text{SSP}2-4.5[\text{consCO}2]}$$ (7)

$$\Delta C_{\text{soleCO}2,\text{SSP}1}=\sum {\text{NBP}}_{\text{SSP}1-4.5}–\sum {\text{NBP}}_{\text{SSP}1-4.5[\text{consCO}2]}$$ (8)

$$\Delta C_{\text{soleCO}2\text{diff}}=\Delta C_{\text{soleCO}2,\text{SSP}2,\text{year}2100}–\Delta C_{\text{soleCO}2,\text{SSP}1,\text{year}2100}$$ (9)

### Changes in the additional carbon sink capacity calculation

The deforestation associated with bioenergy expansion may result in a loss of the additional carbon sink capacity (*29*) because it loses the ability of the replaced forests to accumulate carbon in response to rising CO_2_. We first estimate the sole impact of LULCC (ΔC_LULCC_) as the difference in NBP between two scenarios with the same climate (RCP4.5) but different LULCC. The LULCC effect is further separated depending on whether these two scenarios are driving by constant (ΔC_LULCC,consCO2_; Eqs. 10 and 13 and Fig. 3B) or rising CO_2_ (ΔC_LULCC,risingCO2_; Eqs. 11 and 14 and Fig. 3B). We then calculated the changes in the additional carbon sink capacity by 2100 (ΔC_addition_) as the difference between ΔC_LULCC,risingCO2_ and ΔC_LULCC,consCO2_ in 2100. Equations 12 and 15 are the calculation of ΔC_addition_ in SSP2-4.5 (ΔC_addition,SSP2_, which is −2.4 PgC in Fig. 3B) and SSP1-4.5 (ΔC_addition,SSP1_, which is +2.2 PgC in Fig. 3B), respectively

$$\Delta C_{\text{LULCC},\text{consCO}2,\text{SSP}2}=\sum {\text{NBP}}_{\text{SSP}2-4.5[\text{consCO}2]}–\sum {\text{NBP}}_{\text{RCP}4.5}$$ (10)

$$\Delta C_{\text{LULCC},\text{risingCO}2,\text{SSP}2}=\sum {\text{NBP}}_{\text{SSP}2-4.5}-\sum {\text{NBP}}_{\text{RCP}4.5\;+\;\text{CO}2}$$ (11)

$$\begin{array}{c} \Delta C_{\text{addition},\text{SSP}2}=\Delta C_{\text{LULCC},\text{risingCO}2,\text{SSP}2,\text{year}2100}\\ -\Delta C_{\text{LULCC},\text{consCO}2,\text{SSP}2,\text{year}2100} \end{array}$$ (12)

$$\Delta C_{\text{LULCC},\text{consCO}2,\text{SSP}1}=\sum {\text{NBP}}_{\text{SSP}1-4.5[\text{consCO}2]}-\sum {\text{NBP}}_{\text{RCP}4.5}$$ (13)

$$\Delta C_{\text{LULCC},\text{risingCO}2,\text{SSP}1}=\sum {\text{NBP}}_{\text{SSP}1-4.5}-\sum {\text{NBP}}_{\text{RCP}4.5\;+\;\text{CO}2}$$ (14)

$$\begin{array}{c} \Delta C_{\text{addition},\text{SSP}1}=\Delta C_{\text{LULCC},\text{risingCO}2,\text{SSP}1,\text{year}2100}\\ -\Delta C_{\text{LULCC},\text{consCO}2,\text{SSP}1,\text{year}2100} \end{array}$$ (15)

### Water stress classification considering both water availability and quality

To assess water stress, we consider several physical aspects related to water resources, including both water availability and quality (*19*). In this study, we define severe water stress for the cases when reduction in water availability (runoff) or reduction in water quality due to an increase in nitrogen leaching (soil mineral NO_3_ loss to leaching) is greater than 50% compared to the historical period in the SSP2-4.5 and SSP1-4.5 scenarios. We chose the 50% threshold to be consistent with how previous studies defined severe water scarcity (*59*). Note that the actual water stress for humans or ecosystems would be water available relative to human or plant demands.

Changes in water availability (∆WA) is calculated as the difference of runoff between the future and historical periods

$$\Delta \text{WA}=\frac{{\text{WA}}_{\text{fut}}-{\text{WA}}_{\text{hist}}}{{\text{WA}}_{\text{hist}}}\times 100\%$$ (16)

where WA_fut_ and WA_hist_ are mean annual water availability during 2070–2099 and 1975–2004, respectively.

Changes in nitrogen leaching (∆NL) is calculated as the difference of nitrogen leaching between the historical and future periods

$$\Delta \text{NL}=\frac{{\text{NL}}_{\text{fut}}-{\text{NL}}_{\text{hist}}}{{\text{NL}}_{\text{hist}}}\times 100\%$$ (17)

where NL_fut_ and NL_hist_ are mean annual nitrogen leaching during 2070–2099 and 1975–2004, respectively. Note that increased nitrogen leaching means degraded water quality.

We further classify the severe water stress into four categories, which are water availability limit, water quality limit, both water availability and quality limit, and no limit (Fig. 5), depending upon whether water availability and/or quality have decreased compared to the historical period. Specifically, we define a reduction of 50% in runoff as a proxy for the water availability limit category of water stress, while an increase of 50% in nitrogen leaching as a proxy for the water quality limit category of water stress. These four categories of severe water stress are calculated in Eqs. 18 to 21, respectively

−100%<ΔWA<−50% and ΔNL<50% (18)

ΔWA>−50% and ΔNL>50% (19)

−100%<ΔWA<−50% and ΔNL>50% (20)

ΔWA≥0% and ΔNL≤0 (21)

We calculate how many people will experience severe water stress by 2100 in a grid-based way. First, we extract the projected population (*P*) in SSP2-4.5 and SSP1-4.5 at a 1°/8° resolution over the CONUS from a global dataset (*34*, *35*). Second, we multiply the predicted water stress fraction (*f*_ws_) to the population data in a spatially explicit way. Then, we sum up how many people will live in regions with water limitation over all the grids (Eq. 22).

$$P_{\text{ws}}=\sum_{i=1}^{N}(f_{\text{ws},i}\times P_{i})$$ (22)

where *N* is the number of the grid cells in the study domain, *f*_ws,*i*_ is fraction of land areas under severe water stress in the *i*th grid cell, and *P_i_* is the total projected population in the *i*th grid cell.

## Notes

View/request a protocol for this paper from *Bio-protocol*.

## References

[1] V. Heck, D. Gerten, W. Lucht, A. Popp, Biomass-based negative emissions difficult to reconcile with planetary boundaries. Nat. Clim. Chang. 8, 151–155 (2018).

[2] A. B. Harper, T. Powell, P. M. Cox, J. House, C. Huntingford, T. M. Lenton, S. Sitch, E. Burke, S. E. Chadburn, W. J. Collins, E. Comyn-Platt, V. Daioglou, J. C. Doelman, G. Hayman, E. Robertson, D. van Vuuren, A. Wiltshire, C. P. Webber, A. Bastos, L. Boysen, P. Ciais, N. Devaraju, A. K. Jain, A. Krause, B. Poulter, S. Shu, Land-use emissions play a critical role in land-based mitigation for Paris climate targets. Nat. Commun. 9, 2938 (2018). 30087330 10.1038/s41467-018-05340-z PMC6081380

[3] S. Fuss, J. G. Canadell, G. P. Peters, M. Tavoni, R. M. Andrew, P. Ciais, R. B. Jackson, C. D. Jones, F. Kraxner, N. Nakicenovic, C. L. Quéré, M. R. Raupach, A. Sharifi, P. Smith, Y. Yamagata, COMMENTARY: Betting on negative emissions. Nat. Clim. Chang. 4, 850–853 (2014).

[4] A. Popp, K. Calvin, S. Fujimori, P. Havlik, F. Humpenöder, E. Stehfest, B. L. Bodirsky, J. P. Dietrich, J. C. Doelmann, M. Gusti, T. Hasegawa, P. Kyle, M. Obersteiner, A. Tabeau, K. Takahashi, H. Valin, S. Waldhoff, I. Weindl, M. Wise, E. Kriegler, H. Lotze-Campen, O. Fricko, K. Riahi, D. P. van Vuuren, Land-use futures in the shared socio-economic pathways. Glob. Environ. Chang. 42, 331–345 (2017).

[5] B. W. Griscom, J. Adams, P. W. Ellis, R. A. Houghton, G. Lomax, D. A. Miteva, W. H. Schlesinger, D. Shoch, J. V. Siikamäki, P. Smith, P. Woodbury, C. Zganjar, A. Blackman, J. Campari, R. T. Conant, C. Delgado, P. Elias, T. Gopalakrishna, M. R. Hamsik, M. Herrero, J. Kiesecker, E. Landis, L. Laestadius, S. M. Leavitt, S. Minnemeyer, S. Polasky, P. Potapov, F. E. Putz, J. Sanderman, M. Silvius, E. Wollenberg, J. Fargione, Natural climate solutions. Proc. Natl. Acad. Sci. U.S.A. 114, 11645–11650 (2017). 29078344 10.1073/pnas.1710465114 PMC5676916

[6] D. Huppmann, E. Kriegler, V. Krey, K. Riahi, J. Rogelj, K. Calvin, F. Humpenoeder, A. Popp, S. K. Rose, J. Weyant, N. Bauer, C. Bertram, V. Bosetti, J. Doelman, L. Drouet, J. Emmerling, S. Frank, S. Fujimori, D. Gernaat, A. Grubler, C. Guivarch, M. Haigh, C. Holz, G. Iyer, E. Kato, K. Keramidas, A. Kitous, F. Leblanc, J.-Y. Liu, K. Löffler, G. Luderer, A. Marcucci, D. M. Collum, *S. Mima*, R. D. Sands, F. Sano, J. Strefler, J. Tsutsui, D. Van Vuuren, Z. Vrontisi, M. Wise, R. Zhang, IAMC 1.5°C Scenario Explorer and Data hosted by IIASA (Integrated Assessment Modeling Consortium & International Institute for Applied Systems Analysis, 2019); doi:10.5281/zenodo.3363345.

[7] B. O’Neill, C. Tebaldi, D. van Vuuren, V. Eyring, P. Friedlingstein, G. Hurtt, R. Knutti, E. Kriegler, J.-F. Lamarque, J. Lowe, G. A. Meehl, R. Moss, K. Riahi, B. M. Sanderson, The Scenario Model Intercomparison Project (ScenarioMIP) for CMIP6. Geosci. Model Dev. 9, 3461–3482 (2016).

[8] J. Rogelj, A. Popp, K. V. Calvin, G. Luderer, J. Emmerling, D. Gernaat, S. Fujimori, J. Strefler, T. Hasegawa, G. Marangoni, V. Krey, E. Kriegler, K. Riahi, D. P. van Vuuren, J. Doelman, L. Drouet, J. Edmonds, O. Fricko, M. Harmsen, P. Havlík, F. Humpenöder, E. Stehfest, M. Tavoni, Scenarios towards limiting global mean temperature increase below 1.5°C. Nat. Clim. Chang. 8, 325–332 (2018).

[9] B. C. O’Neill, E. Kriegler, K. L. Ebi, E. Kemp-Benedict, K. Riahi, D. S. Rothman, B. J. van Ruijven, D. P. van Vuuren, J. Birkmann, K. Kok, M. Levy, W. Solecki, The roads ahead: Narratives for shared socioeconomic pathways describing world futures in the 21st century. Glob. Environ. Chang. 42, 169–180 (2017).

[10] K. Riahi, D. P. van Vuuren, E. Kriegler, J. Edmonds, B. C. O’Neill, S. Fujimori, N. Bauer, K. Calvin, R. Dellink, O. Fricko, W. Lutz, A. Popp, J. C. Cuaresma, S. KC, M. Leimbach, L. Jiang, T. Kram, S. Rao, J. Emmerling, K. Ebi, T. Hasegawa, P. Havlik, F. Humpenöder, L. A. da Silva, S. Smith, E. Stehfest, V. Bosetti, J. Eom, D. Gernaat, T. Masui, J. Rogelj, J. Strefler, L. Drouet, V. Krey, G. Luderer, M. Harmsen, K. Takahashi, L. Baumstark, J. C. Doelman, M. Kainuma, Z. Klimont, G. Marangoni, H. Lotze-Campen, M. Obersteiner, A. Tabeau, M. Tavoni, The Shared Socioeconomic Pathways and their energy, land use, and greenhouse gas emissions implications: An overview. Glob. Environ. Chang. 42, 153–168 (2017).

[11] M. Chen, C. R. Vernon, N. T. Graham, M. Hejazi, M. Huang, Y. Cheng, K. Calvin, Global land use for 2015–2100 at 0.05° resolution under diverse socioeconomic and climate scenarios. Sci. Data 7, 320 (2020). 33009403 10.1038/s41597-020-00669-x PMC7532189

[12] P. Withey, C. Johnston, J. Guo, Quantifying the global warming potential of carbon dioxide emissions from bioenergy with carbon capture and storage. Renew. Sustain. Energy Rev. 115, 109408 (2019).

[13] M. I. Hejazi, N. Voisin, L. Liu, L. M. Bramer, D. C. Fortin, J. E. Hathaway, M. Huang, P. Kyle, L. R. Leung, H.-Y. Li, Y. Liu, P. L. Patel, T. C. Pulsipher, J. S. Rice, T. K. Tesfa, C. R. Vernon, Y. Zhou, 21st century United States emissions mitigation could increase water stress more than the climate change it is mitigating. Proc. Natl. Acad. Sci. U.S.A. 112, 10635–10640 (2015). 26240363 10.1073/pnas.1421675112 PMC4553779

[14] F. Creutzig, A. Popp, R. Plevin, G. Luderer, J. Minx, O. Edenhofer, Reconciling top-down and bottom-up modelling on future bioenergy deployment. Nat. Clim. Chang. 2, 320–327 (2012).

[15] D. M. Lapola, R. Schaldach, J. Alcamo, A. Bondeau, J. Koch, C. Koelking, J. A. Priess, Indirect land-use changes can overcome carbon savings from biofuels in Brazil. Proc. Natl. Acad. Sci. U.S.A. 107, 3388–3393 (2020). 10.1073/pnas.0907318107 PMC2840431 20142492

[16] P. V. V. Le, P. Kumar, D. T. Drewry, Implications for the hydrologic cycle under climate change due to the expansion of bioenergy crops in the Midwestern United States. Proc. Natl. Acad. Sci. U.S.A. 108, 15085–15090 (2011). 21876137 10.1073/pnas.1107177108 PMC3174653

[17] F. Stenzel, P. Greve, W. Lucht, S. Tramberend, Y. Wada, D. Gerten, Irrigation of biomass plantations may globally increase water stress more than climate change. Nat. Commun. 12, 1512 (2021). 33686076 10.1038/s41467-021-21640-3 PMC7940422

[18] E. Sinha, A. M. Michalak, K. V. Calvin, P. J. Lawrence, Societal decisions about climate mitigation will have dramatic impacts on eutrophication in the 21st century. Nat. Commun. 10, 939 (2019). 30808880 10.1038/s41467-019-08884-w PMC6391408

[19] M. T. H. van Vliet, M. Flörke, Y. Wada, Quality matters for water scarcity. Nat. Geosci. 10, 800–802 (2017).

[20] J. L. Field, T. L. Richard, E. A. H. Smithwick, H. Cai, M. S. Laser, D. S. Le Bauer, S. P. Long, K. Paustian, Z. Qin, J. J. Sheehan, P. Smith, M. Q. Wang, L. R. Lynd, Robust paths to net greenhouse gas mitigation and negative emissions via advanced biofuels. Proc. Natl. Acad. Sci. U.S.A. 117, 21968–21977 (2020). 32839342 10.1073/pnas.1920877117 PMC7486778

[21] R. Righelato, D. V. Spracklen, Carbon mitigation by biofuels or by saving and restoring forests? Science 317, 902 (2007). 17702929 10.1126/science.1141361

[22] K. Calvin, P. Patel, L. Clarke, G. Asrar, B. Bond-Lamberty, R. Y. Cui, A. D. Vittorio, K. Dorheim, J. Edmonds, C. Hartin, M. Hejazi, R. Horowitz, G. Iyer, P. Kyle, S. Kim, R. Link, H. Mcjeon, S. J. Smith, A. Snyder, S. Waldhoff, M. Wise, GCAM v5.1: Representing the linkages between energy, water, land, climate, and economic systems. Geosci. Model Dev. 12, 677–698 (2019).

[23] M. Chen, C. R. Vernon, M. Huang, K. V. Calvin, I. P. Kraucunas, Calibration and analysis of the uncertainty in downscaling global land use and land cover projections from GCAM using Demeter (v1.0.0). Geosci. Model Dev. 12, 1753–1764 (2019).

[24] Y. Cheng, M. Huang, M. Chen, K. Guan, C. Bernacchi, B. Peng, Z. Tan, Parameterizing perennial bioenergy crops in version 5 of the community land model based on site-level observations in the central Midwestern United States. J. Adv. Model. Earth Syst. 12, e2019MS001719 (2020).

[25] B. C. O’Neill, E. Kriegler, K. Riahi, K. L. Ebi, S. Hallegatte, T. R. Carter, R. Mathur, D. P. van Vuuren, A new scenario framework for climate change research: The concept of shared socioeconomic pathways. Clim. Change 122, 387–400 (2014).

[26] J. S. Sperry, M. D. Venturas, H. N. Todd, A. T. Trugman, W. R. L. Anderegg, Y. Wang, X. Tai, The impact of rising CO_2_ and acclimation on the response of US forests to global warming. Proc. Natl. Acad. Sci. U.S.A. 116, 25734–25744 (2019). 31767760 10.1073/pnas.1913072116 PMC6926066

[27] E. A. Ainsworth, A. Rogers, The response of photosynthesis and stomatal conductance to rising [CO_2_]: Mechanisms and environmental interactions. Plant Cell Environ. 30, 258–270 (2007). 17263773 10.1111/j.1365-3040.2007.01641.x

[28] Y. Liu, S. Piao, T. Gasser, P. Ciais, H. Yang, H. Wang, T. F. Keenan, M. Huang, S. Wan, J. Song, K. Wang, I. A. Janssens, J. Peñuelas, C. Huntingford, X. Wang, M. Altaf Arain, Y. Fang, J. B. Fisher, M. Huang, D. N. Huntzinger, A. Ito, A. K. Jain, J. Mao, A. M. Michalak, C. Peng, B. Poulter, C. Schwalm, X. Shi, H. Tian, Y. Wei, N. Zeng, Q. Zhu, T. Wang, Field-experiment constraints on the enhancement of the terrestrial carbon sink by CO_2_ fertilization. Nat. Geosci. 12, 809–814 (2019).

[29] J. Pongratz, C. H. Reick, R. A. Houghton, J. I. House, Terminology as a key uncertainty in net land use and land cover change carbon flux estimates. Earth Syst. Dynam. 5, 177–195 (2014).

[30] S. L. Lewis, C. E. Wheeler, E. T. A. Mitchard, A. Koch, Restoring natural forests is the best way to remove atmospheric carbon. Nature 568, 25–28 (2019). 30940972 10.1038/d41586-019-01026-8

[31] B. Zhu, M. Huang, Y. Cheng, X. Xie, Y. Liu, G. Bisht, X. Chen, Impact of vegetation physiology and phenology on watershed hydrology in a semiarid watershed in the Pacific Northwest in a changing climate. Water Resour. Res. 57, e2020WR028394 (2021).

[32] T. E. Johnson, J. B. Butcher, A. Parker, C. P. Weaver, Investigating the sensitivity of U.S. streamflow and water quality to climate change: U.S. EPA Global Change Research Program’s 20 watersheds project. J. Water Resour. Plan. Manag. 138, 453–464 (2012).

[33] Y. Gao, L. Ruby Leung, J. Lu, Y. Liu, M. Huang, Y. Qian, Robust spring drying in the southwestern U.S. and seasonal migration of wet/dry patterns in a warmer climate. Geophys. Res. Lett. 41, 1745–1751 (2014).

[34] B. Jones, B. C. O’Neill, Spatially explicit global population scenarios consistent with the Shared Socioeconomic Pathways. Environ. Res. Lett. 11, 084003 (2016).

[35] B. Jones, B. C. O’Neill, *Global One-Eighth Degree Population Base Year and Projection Grids Based on the Shared Socioeconomic Pathways, Revision 01* (Palisades, NY: NASA Socioeconomic Data and Applications Center, 2020).

[36] E. Sinha, A. M. Michalak, V. Balaji, Eutrophication will increase during the 21st century as a result of precipitation changes. Science 357, 405–408 (2017). 28751610 10.1126/science.aan2409

[37] Y. L. Page, T. O. West, R. Link, P. Patel, Downscaling land use and land cover from the Global Change Assessment Model for coupling with Earth system models. Geosci. Model Dev. 9, 3055–3069 (2016).

[38] D. M. Lawrence, R. A. Fisher, C. D. Koven, K. W. Oleson, S. C. Swenson, G. Bonan, N. Collier, B. Ghimire, L. van Kampenhout, D. Kennedy, E. Kluzek, P. J. Lawrence, F. Li, H. Li, D. Lombardozzi, W. J. Riley, W. J. Sacks, M. Shi, M. Vertenstein, W. R. Wieder, C. Xu, A. A. Ali, A. M. Badger, G. Bisht, M. van den Broeke, M. A. Brunke, S. P. Burns, J. Buzan, M. Clark, A. Craig, K. Dahlin, B. Drewniak, J. B. Fisher, M. Flanner, A. M. Fox, P. Gentine, F. Hoffman, G. Keppel-Aleks, R. Knox, S. Kumar, J. Lenaerts, L. Ruby Leung, W. H. Lipscomb, Y. Lu, A. Pandey, J. D. Pelletier, J. Perket, J. T. Randerson, D. M. Ricciuto, B. M. Sanderson, A. Slater, Z. M. Subin, J. Tang, R. Q. Thomas, M. V. Martin, X. Zeng, The community land model version 5: Description of new features, benchmarking, and impact of forcing uncertainty. J. Adv. Model. Earth Syst. 11, 4245–4287 (2019).

[39] D. L. Lombardozzi, Y. Lu, P. J. Lawrence, D. M. Lawrence, S. Swenson, K. W. Oleson, W. R. Wieder, E. A. Ainsworth, Simulating agriculture in the community land model version 5. Eur. J. Vasc. Endovasc. Surg. 125, e2019JG005529 (2020).

[40] W. Li, P. Ciais, M. Han, Q. Zhao, J. Chang, D. S. Goll, L. Zhu, J. Wang, Bioenergy crops for low warming targets require half of the present agricultural fertilizer use. Environ. Sci. Technol. 55, 10654–10661 (2021). 34288664 10.1021/acs.est.1c02238

[41] E. Monier, S. Paltsev, A. Sokolov, Y.-H. Henry Chen, X. Gao, Q. Ejaz, E. Couzo, C. Adam Schlosser, S. Dutkiewicz, C. Fant, J. Scott, D. Kicklighter, J. Morris, H. Jacoby, R. Prinn, M. Haigh, Toward a consistent modeling framework to assess multi-sectoral climate impacts. Nat. Commun. 9, 660 (2018). 29440736 10.1038/s41467-018-02984-9 PMC5811603

[42] K. Calvin, M. Wise, L. Clarke, J. Edmonds, P. Kyle, P. Luckow, A. Thomson, Implications of simultaneously mitigating and adapting to climate change: Initial experiments using GCAM. Clim. Change 117, 545–560 (2013).

[43] W. Collins, A. Craig, J. Truesdale, A. Vittorio, A. D. Jones, B. Bond-Lamberty, K. Calvin, J. Edmonds, S. H. Kim, A. Thomson, P. Patel, Y. Zhou, J. Mao, X. Shi, P. Thornton, L. Chini, G. Hurtt, The integrated Earth system model version 1: Formulation and functionality. Geosci. Model Dev. 8, 2203–2219 (2015).

[44] P. E. Thornton, K. Calvin, A. D. Jones, A. V. Di Vittorio, B. Bond-Lamberty, L. Chini, X. Shi, J. Mao, W. D. Collins, J. Edmonds, A. Thomson, J. Truesdale, A. Craig, M. L. Branstetter, G. Hurtt, Biospheric feedback effects in a synchronously coupled model of human and Earth systems. Nat. Clim. Chang. 7, 496–500 (2017).

[45] T. O. West, Y. L. Page, M. Huang, J. Wolf, A. M. Thomson, Downscaling global land cover projections from an integrated assessment model for use in regional analyses: Results and evaluation for the US from 2005 to 2095. Environ. Res. Lett. 9, 064004 (2014).

[46] N. T. Graham, M. I. Hejazi, M. Chen, E. G. R. Davies, J. A. Edmonds, S. H. Kim, S. W. D. Turner, X. Li, C. R. Vernon, K. Calvin, F. Miralles-Wilhelm, L. Clarke, P. Kyle, R. Link, P. Patel, A. C. Snyder, M. A. Wise, Humans drive future water scarcity changes across all Shared Socioeconomic Pathways. Environ. Res. Lett. 15, 014007 (2020).

[47] D. M. Lawrence, R. A. Fisher, C. D. Koven, K. W. Oleson, S. C. Swenson, G. Bonan, N. Collier, B. Ghimire, L. van Kampenhout, D. Kennedy, E. Kluzek, P. J. Lawrence, F. Li, H. Li, D. Lombardozzi, W. J. Riley, W. J. Sacks, M. Shi, M. Vertenstein, W. R. Wieder, C. Xu, A. A. Ali, A. M. Badger, G. Bisht, M. van den Broeke, M. A. Brunke, S. P. Burns, J. Buzan, M. Clark, A. Craig, K. Dahlin, B. Drewniak, J. B. Fisher, M. Flanner, A. M. Fox, P. Gentine, F. Hoffman, G. Keppel-Aleks, R. Knox, S. Kumar, J. Lenaerts, L. Ruby Leung, W. H. Lipscomb, Y. Lu, A. Pandey, J. D. Pelletier, J. Perket, J. T. Randerson, D. M. Ricciuto, B. M. Sanderson, A. Slater, Z. M. Subin, J. Tang, R. Quinn Thomas, M. V. Martin, Xubin Zeng, “Technical description of version 5.0 of the Community Land Model (CLM)” (NCAR/TN-478+STR NCAR Technical Note 350, 2018); doi:10.5065/D6RR1W7M.

[48] A. A. Ali, C. Xu, A. Rogers, R. A. Fisher, S. D. Wullschleger, N. G. McDowell, E. Massoud, J. Vrugt, J. Muss, J. B. Fisher, P. B. Reich, C. J. Wilson, A global scale mechanistic model of photosynthetic capacity (LUNA V1.0). Geosci. Model Dev. 9, 587–606 (2016).

[49] R. A. Fisher, W. R. Wieder, B. M. Sanderson, C. D. Koven, K. W. Oleson, C. Xu, J. B. Fisher, M. Shi, A. P. Walker, D. M. Lawrence, Parametric controls on vegetation responses to biogeochemical forcing in the CLM5. J. Adv. Model. Earth Syst. 11, 2879–2895 (2019).

[50] V. Arora, A. Katavouta, R. Williams, C. Jones, V. Brovkin, P. Friedlingstein, J. Schwinger, L. Bopp, O. Boucher, P. Cadule, M. Chamberlain, J. Christian, C. Delire, R. Fisher, T. Hajima, T. Ilyina, E. Joetzjer, M. Kawamiya, C. Koven, J. Krasting, R. Law, D. Lawrence, A. Lenton, K. Lindsay, J. Pongratz, T. Raddatz, R. Séférian, K. Tachiiri, J. Tjiputra, A. Wiltshire, T. Wu, T. Ziehn, Carbon-concentration and carbon-climate feedbacks in CMIP6 models and their comparison to CMIP5 models. Biogeosciences 17, 4173–4222 (2020).

[51] W. R. Wieder, D. M. Lawrence, R. A. Fisher, G. B. Bonan, S. J. Cheng, C. L. Goodale, A. S. Grandy, C. D. Koven, D. L. Lombardozzi, K. W. Oleson, R. Q. Thomas, Beyond static benchmarking: Using experimental manipulations to evaluate land model assumptions. Global Biogeochem. Cycles 33, 1289–1309 (2019). 31894175 10.1029/2018GB006141 PMC6919943

[52] V. Masson-Delmotte, P. Zhai, A. Pirani, S. L. Connors, C. Péan, S. Berger, N. Caud, Y. Chen, L. Goldfarb, M. I. Gomis, M. Huang, K. Leitzell, E. Lonnoy, J. B. R. Matthews, T. K. Maycock, T. Waterfield, O. Yelekçi, R. Yu, B. Zhou., IPCC, 2021: Climate Change 2021: The Physical Science Basis. Contribution of Working Group I to the Sixth Assessment Report of the Intergovernmental Panel on Climate Change (Cambridge University Press, 2021).

[R53] B. Zhu, M. Huang, Y. Cheng, X. Xie, Y. Liu, X. Zhang, G. Bisht, X. Chen, J. Missik, H. Liu, Effects of irrigation on water, carbon, and nitrogen budgets in a semiarid watershed in the Pacific Northwest: A modeling study. J. Adv. Model. Earth Syst. 12, e2019MS001953 (2020).

[54] D. L. Lombardozzi, Y. Lu, P. J. Lawrence, D. M. Lawrence, S. Swenson, K. W. Oleson, W. R. Wieder, E. A. Ainsworth, Simulating agriculture in the Community Land Model version 5. J. Chem. Inf. Model. 53, (2013).

[55] P. Lawrence, T. N. Chase, Representing a new MODIS consistent land surface in the Community Land Model (CLM 3.0). J. Geophys. Res. 112, G01023 (2007).

[56] Y. Cheng, M. Huang, B. Zhu, G. Bisht, T. Zhou, Y. Liu, F. Song, X. He, Validation of the community land model version 5 over the contiguous United States (CONUS) using in situ and remote sensing data sets. J. Geophys. Res. Atmos. 2, 1–27 (2021).

[57] M. G. R. Cannell, Carbon sequestration and biomass energy offset: Theoretical, potential and achievable capacities globally, in Europe and the UK. Biomass Bioenergy 24, 97–116 (2003).

[58] J. C. Clifton-Brown, P. F. Stampfl, M. B. Jones, *Miscanthus* biomass production for energy in Europe and its potential contribution to decreasing fossil fuel carbon emissions. Glob. Chang. Biol. 10, 509–518 (2004).

[59] M. M. Mekonnen, A. Y. Hoekstra, Four billion people facing severe water scarcity. Sci. Adv. 2, e1500323 (2016). 26933676 10.1126/sciadv.1500323 PMC4758739

