# Supplementary material for: Future bioenergy expansion could alter carbon sequestration potential and exacerbate water stress in the United States
Source: Sci Adv. 2022 May 4;8(18):eabm8237. doi: 10.1126/sciadv.abm8237 (PMC11633087; doi:10.1126/sciadv.abm8237)
Supplement: Supplementary file 1 — Figs. S1 to S9 Tables S1 and S2 [file sciadv.abm8237_sm.pdf]

Science Advances
AAAS

Supplementary Materials for

Future bioenergy expansion could alter carbon sequestration potential and exacerbate water stress in the United States

Yanyan Cheng*, Maoyi Huang, David M. Lawrence, Katherine Calvin, Danica L. Lombardozzi, Eva Sinha, Ming Pan, Xiaogang He*

*Corresponding author. Email: yanyan.cheng@nus.edu.sg (Y.C.); hexg@nus.edu.sg (X.H.)

Published 4 May 2022, *Sci. Adv.* **8**, eabm8237 (2022)
DOI: 10.1126/sciadv.abm8237

This PDF file includes:

Figs. S1 to S9
Tables S1 and S2

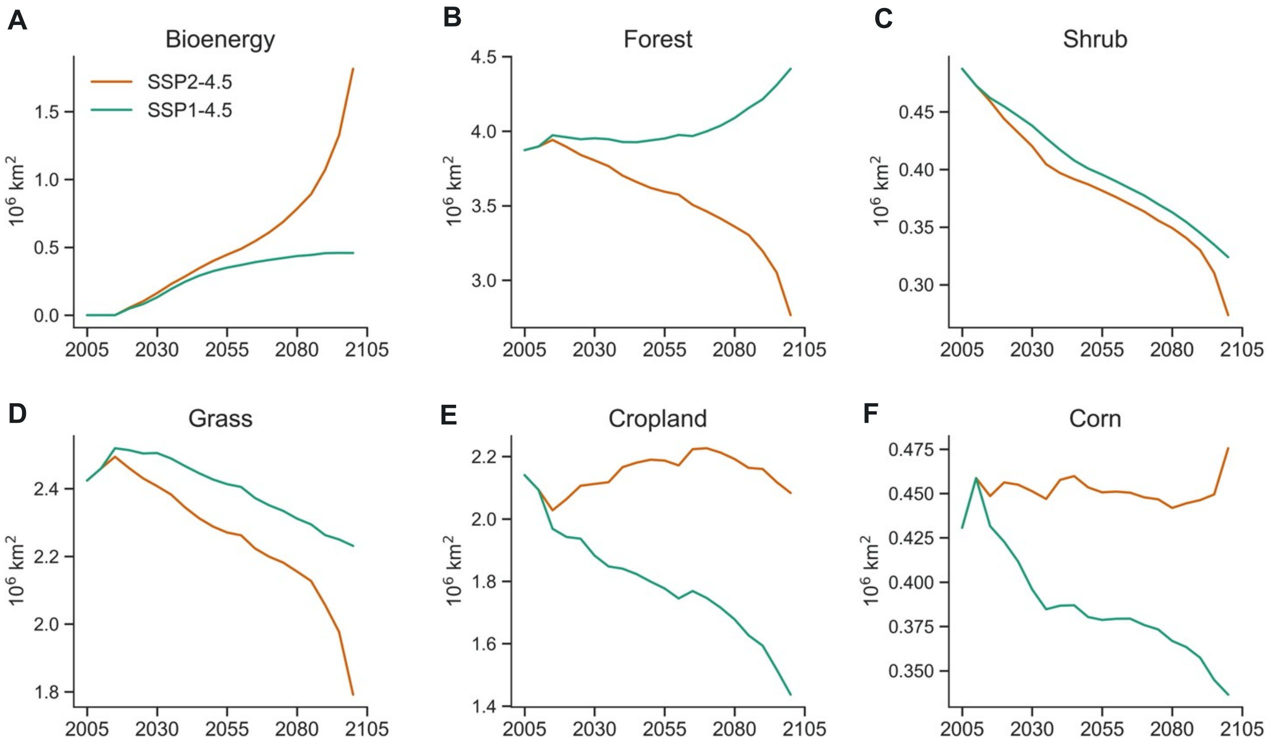

Fig. S1: Changes in total areas of bioenergy crops (**A**), forest (**B**), shrub (**C**), grass (**D**), cropland (**E**), and corn (**F**) over the CONUS from 2005-2100 in the SSP2-4.5 (orange color, primary bioenergy expansion) and SSP1-4.5 (green color, primary reforestation) scenarios. Here the cropland in **E** includes areas of corn, soybean, wheat, cotton, rice, sugarcane, and C3 unmanaged crops.

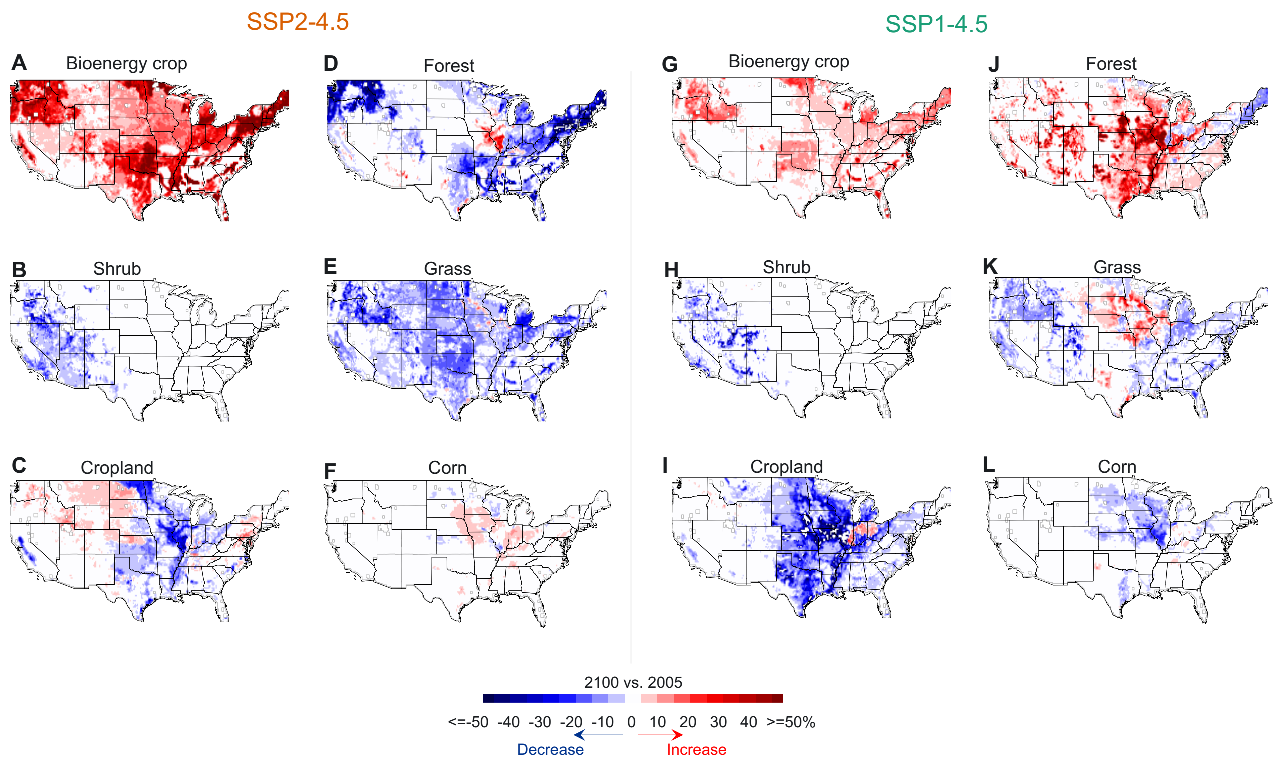

Fig. S2: Changes in areas of bioenergy crops (**A** and **G**), shrub (**B** and **H**), cropland (**C** and **I**), forest (**D** and **J**), grass (**E** and **K**), and corn (**F** and **L**) in SSP2-4.5 (**A-F**, left panel) and SSP1-4.5 (**G-L**, right panel) over the CONUS. Changes are shown as the difference between the year 2100 and 2005. Here the cropland in **C** and **I** includes areas of corn, soybean, wheat, cotton, rice, sugarcane, and C3 unmanaged crops.

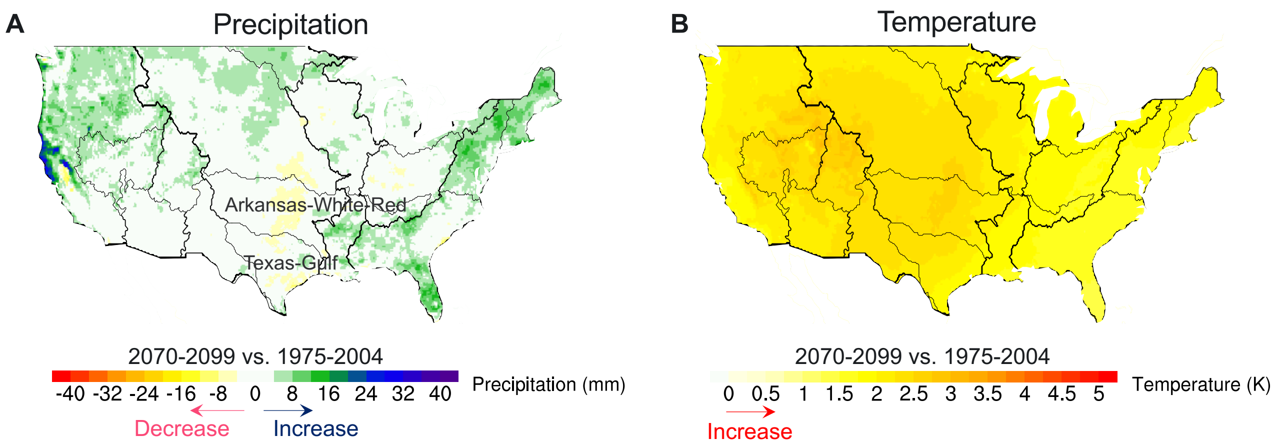

Fig. S3: Changes in mean annual (**A**) precipitation and (**B**) temperature in RCP4.5 by the end-of-the-century (2070-2099) relative to the historical period (1975-2004) over the CONUS.

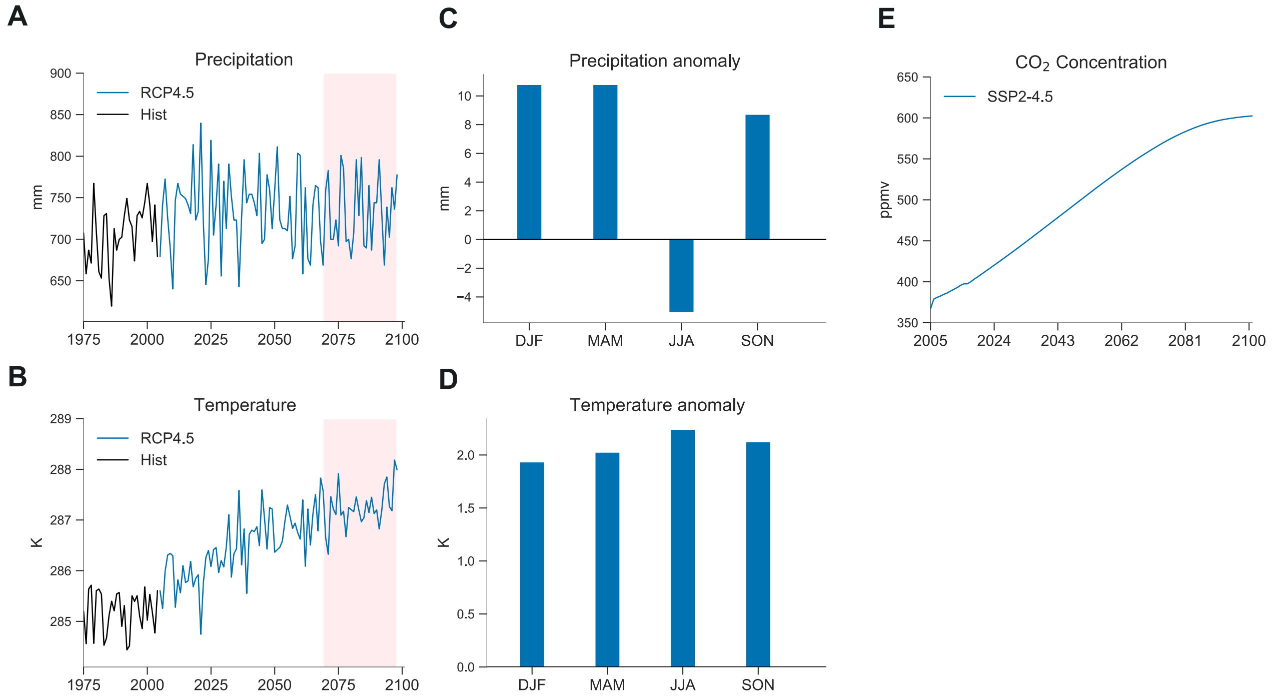

Fig. S4: Annual (**A**) precipitation and (**B**) temperature from 1975-2100 in RCP4.5, and seasonal (**C**) precipitation anomaly and (**D**) temperature anomaly between the end-of-the-century (2070-2099, light red background) and the historical period in RCP4.5, and (**E**) CO_2 concentration in SSP2-4.5 from 2005 to 2100.

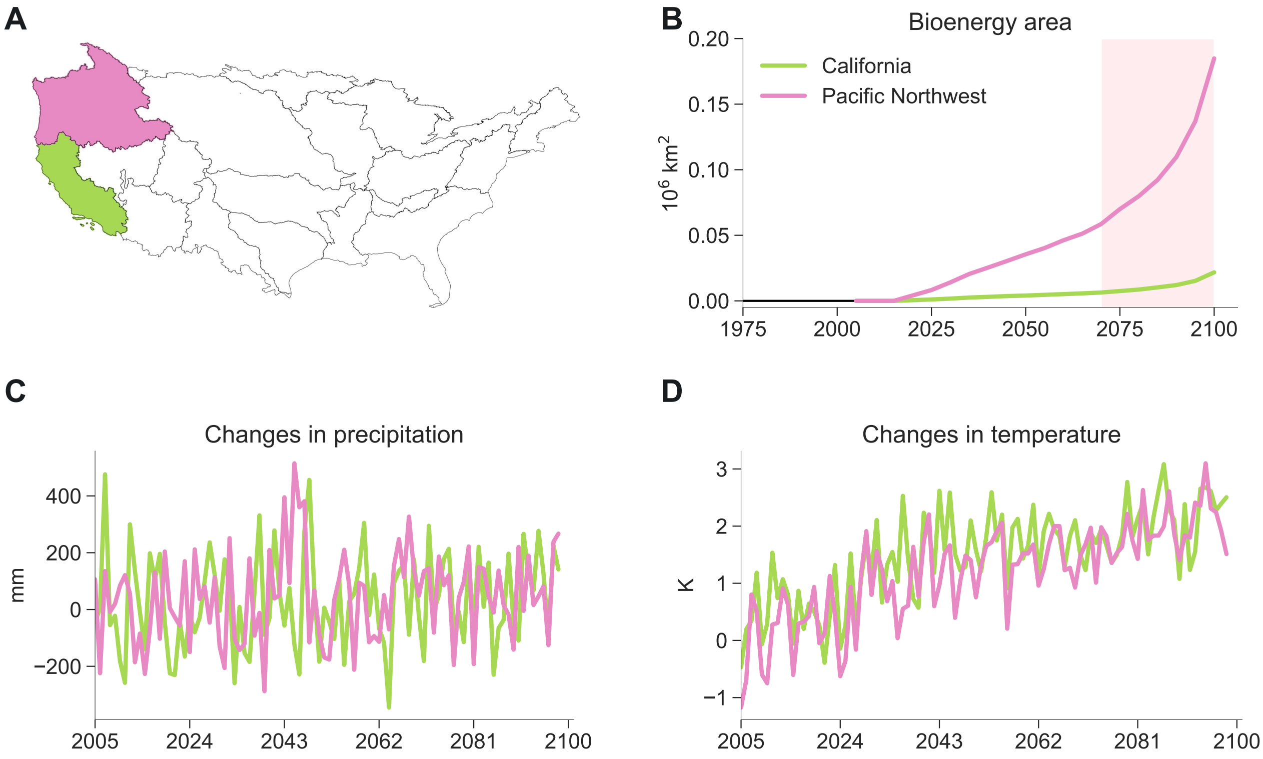

Fig. S5: (**A**) Boundaries for the California (low bioenergy expansion) and Pacific Northwest (high bioenergy expansion) regions, (**B**) Changes in plantation areas of bioenergy crops from 1975-2100 in SSP2-4.5 for the two regions, and changes in mean annual (**C**) precipitation and (**D**) temperature during 2005-2100 compared to the historical period.

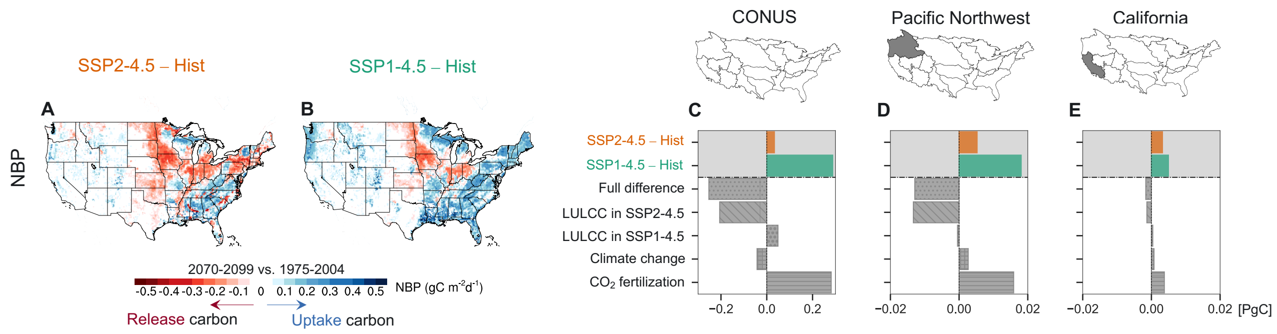

Fig. S6: Changes in climate and land uses result in large differences in NBP over the CONUS by the end of the century. Changes are shown as the absolute differences between the end-of-the-century (2070–2099) relative to the historical period (1975–2004) for the (**A**) SSP2-4.5 (first column) and (**B**) SSP1-4.5 (second column) scenarios. Changes in NBP for (**C**) CONUS, (**E**) Pacific Northwest region (high bioenergy expansion), and (**E**) California region (low bioenergy expansion) in the two scenarios and attributions to individual components including full difference between SSP2-4.5 and SSP1-4.5, LULCCs in SSP2-4.5 and SSP1-4.5, climate change, and CO_2 fertilization.

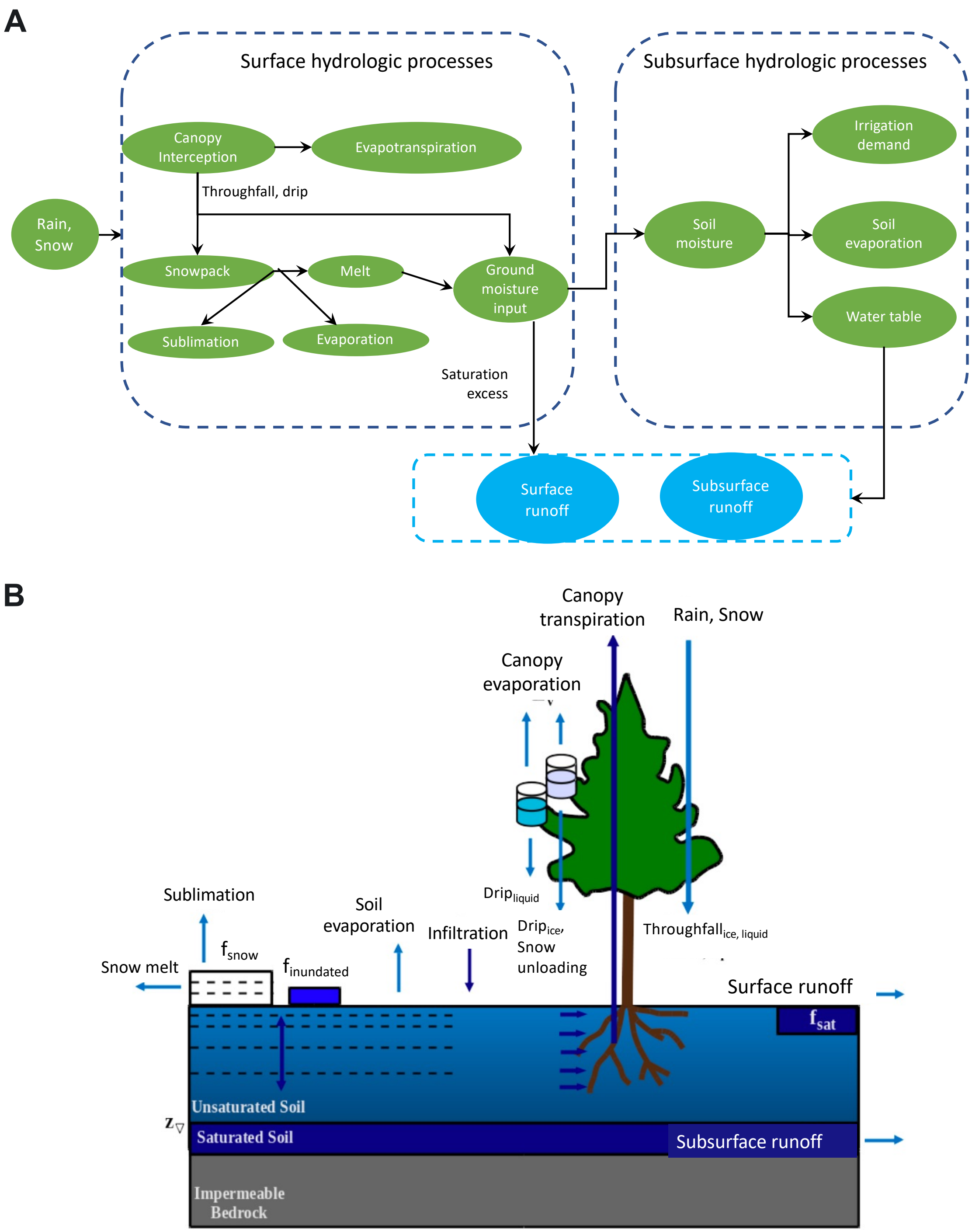

Fig. S7: The surface and subsurface hydrological processes represented in CLM5. **B** is modified from Lawrence et al., 2019 (Lawrence, D. *et al.* Technical description of version 5.0 of the Community Land Model (CLM). *NCAR/TN-478+STR NCAR Tech. Note* 350 (2018) doi:10.5065/D6RR1W7M).

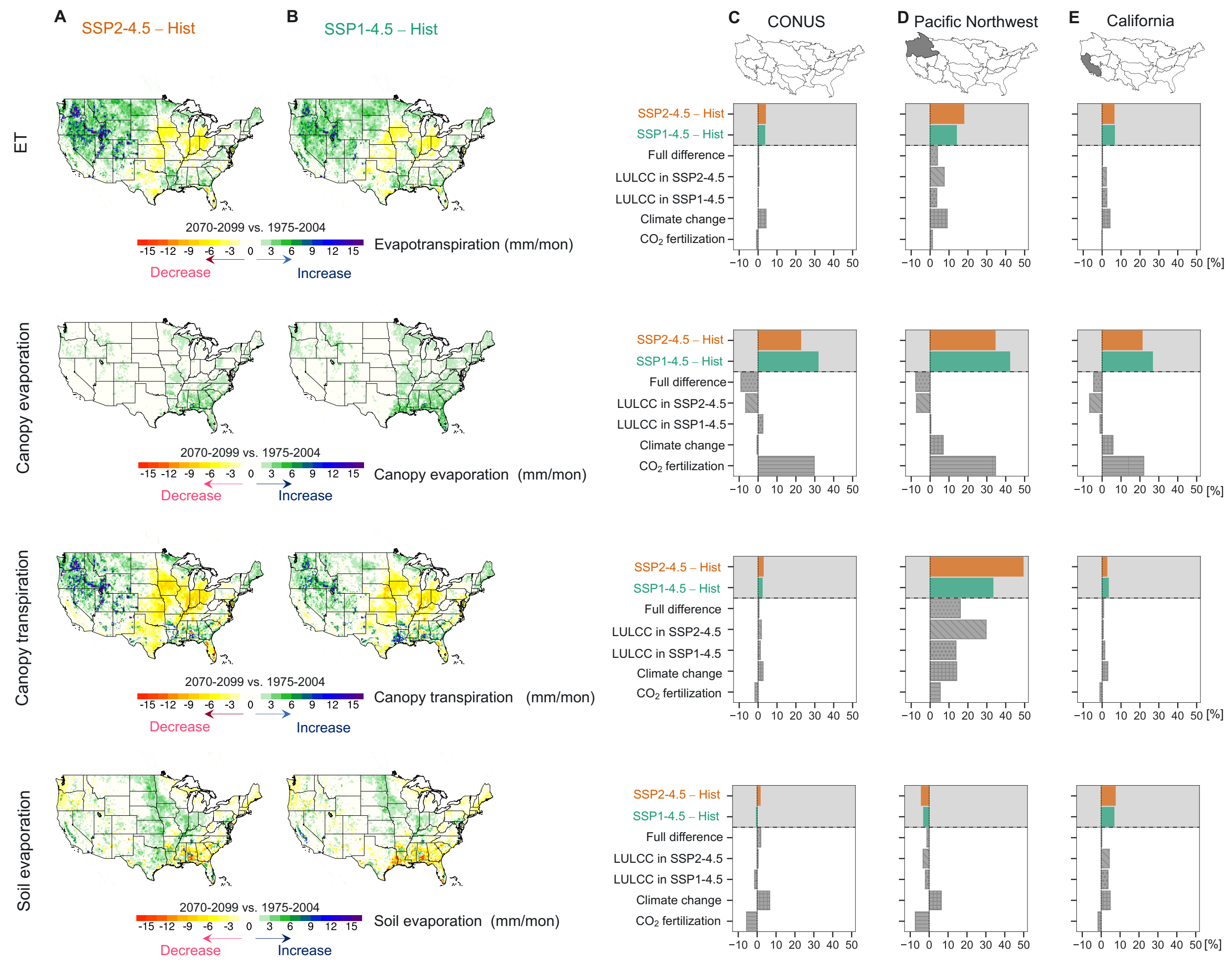

Fig. S8: Same as **Fig. S6**, but for evapotranspiration (ET, first row) and its three components: canopy evaporation (second row), canopy transpiration (third row), and soil evaporation (fourth row).

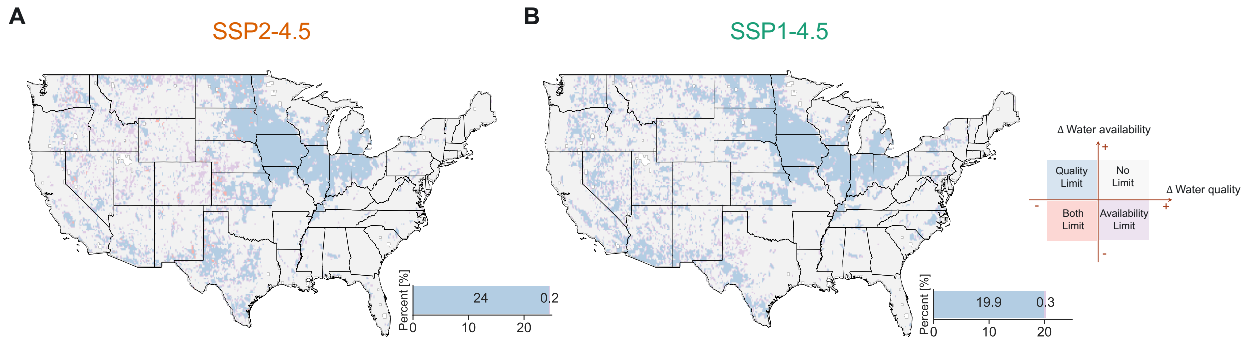

Fig. S9: Water stress caused by the combined effects of climate change, CO_2 fertilization, and LULCC over the CONUS in (**A**) SSP2-4.5 and (**B**) SSP1-4.5.

Table S1: Scenario overview.

Name of simulations	Climate forcing	LULCC	CO_2 concentration	Period	Short description
SSP2-4.5	RESM RCP4.5	Transient (SSP2-4.5)	Transient (SSP2-4.5)	2005-2100	Climate change + CO_2 fertilization + LULCC. This is the combination of the SSP2 middle of the road pathway with the RCP4.5 climate outcome, driven by transient *rising* CO_2. Land use changes are driven by *bioenergy expansion*.
SSP1-4.5	RESM RCP4.5	Transient (SSP1-4.5)	Transient (SSP2-4.5)	2005-2100	Climate change + CO_2 fertilization + LULCC. This is the combination of the SSP1 sustainable development with the RCP4.5 climate outcome, driven by transient *rising* CO_2. Only limited bioenergy expansion and land use changes are mainly driven by *reforestation*.
SSP2-4.5[consCO2]	RESM RCP4.5	Transient (SSP2-4.5)	Constant (367 ppmv)	2005-2100	Climate change + constant CO_2 + LULCC. This is the combination of the SSP2 middle of the road pathway with the RCP4.5 climate outcome, driven by *constant* CO_2. Land use changes are driven by *bioenergy expansion*.
SSP1-4.5[consCO2]	RESM RCP4.5	Transient (SSP1-4.5)	Constant (367 ppmv)	2005-2100	Climate change + constant CO_2 + LULCC. This is the combination of the SSP1 sustainable development with the RCP4.5 climate outcome, driven by *constant* CO_2. Only limited bioenergy expansion and land use changes are mainly driven by *reforestation*.
Hist	RESM historical	year 2000	Constant (367 ppmv)	1975-2004	Historical condition, control run
RCP4.5	RESM RCP4.5	year 2000	Constant (367 ppmv)	2005-2100	Climate change only
RCP4.5+CO_2	RESM RCP4.5	year 2000	Transient (SSP2-4.5)	2005-2100	Climate change + CO_2 fertilization

Note: LULCC=land use and land cover changes, RESM=regional Earth System model, RCP=representative concentration pathways, SSP=Shared Socio-economic Pathways.

Table S2: Attribution analysis of bioenergy expansion, LULCC, climate change, and CO_2 fertilization.

Individual factor	Calculation
Bioenergy expansion and reforestation (i.e., the full difference between SSP2-4.5 and SSP1-4.5)	SSP2-4.5 versus SSP1-4.5
Total LULCC driven by primary bioenergy expansion	SSP2-4.5 versus RCP4.5+CO_2
Total LULCC driven by primary reforestation	SSP1-4.5 versus RCP4.5+CO_2
Climate change	RCP4.5 versus HIST
CO_2 fertilization	RCP4.5+CO_2 versus RCP4.5

Note: the scenario names are given in Table S1.
